# *Parabacteroides goldsteinii* and its metabolite 7-KLCA attenuate endometriosis via TGR5 to reprogram macrophages by modulating the PPARγ/GPR132 axis

**DOI:** 10.1038/s41522-026-01017-4

**Published:** 2026-05-22

**Authors:** Yun Chen, Yuqing Qiu, Feifei Pan, Yanqin Zheng, Jingyao Wang, Kunxiang Gong, Lirong Guo, Yiming Song, Zixin Tao, Kun Shi

**Affiliations:** 1 Department of Gynecology and Obstetrics, Guangzhou Women and Children’s Medical Center, Guangzhou Medical University, Guangzhou, China; 2 Institute of Reproductive Health and Perinatology, Guangzhou Women and Children’s Medical Center, Guangzhou Medical University, Guangzhou, China; 3 Department of Gynecology and Obstetrics, Guangzhou First People’s Hospital, South China University of Technology, Guangzhou, China

**Keywords:** Diseases, Immunology, Microbiology

## Abstract

Endometriosis (EMS) remains understudied in effective management strategies. The interplay between macrophage dysfunction and microbiota-derived immune signals emerges as a potential mechanism in EMS pathogenesis, suggesting its relevance for future therapeutic exploration. In this study, we established mouse models to demonstrate that gut microbiota modulated EMS development. Integrated microbial and metabolomic profiling identified *Parabacteroides goldsteinii* (Pg) as a promising probiotic candidate, whose downstream metabolite 7-ketolithocholic acid (7-KLCA) exhibiting therapeutic efficacy in ameliorating EMS phenotypes upon supplementation. Mechanistically, Pg reshapes its bile acid (BA) metabolism to elevate 7-KLCA. This bioactive metabolite acts via the receptor TGR5 to suppress PPARγ expression and activate GPR132, thereby enhancing efferocytosis and promoting M1 macrophage polarization. These findings uncover a gut-metabolite-immune regulatory axis through which Pg reprograms macrophage function to restrain EMS progression, offering a potential microbial perspective for this chronic condition.

## Introduction

EMS is a chronic, estrogen-dependent inflammatory condition characterized by the ectopic growth of endometrial-like tissue, affecting approximately 10% of women of reproductive age^1–3^. Patients commonly experience dysmenorrhea, infertility, and chronic pelvic pain, which contribute to significant physical and psychological burdens^4^. Despite its high prevalence, the pathogenesis of EMS remains incompletely understood, and current therapeutic options are limited by high recurrence rates and systemic side effects^5^.

Growing evidence implicates macrophage-driven mechanisms in the pathogenesis of endometriosis^6–9^. Initially, proinflammatory M1 macrophages are often predominant and appear to attempt elimination of ectopic lesions. Subsequent shifts toward M2-polarized phenotypes may contribute to an immunosuppressive microenvironment, promoting angiogenesis and adhesion formation while impairing immune surveillance through effector cell dysfunction^10–12^. This dysfunctional polarization points to macrophages as potential drivers of EMS pathology.

The role of the gut microbiota (GM) in endometriosis (EMS) is actively investigated, yet compositional studies report conflicting findings^13–16^. This inconsistency underscores the necessity for functional evidence. Although antibiotic-mediated GM perturbation can influence EMS phenotypes, the definitive causal links and mechanisms remain elusive^17,18^. In particular, it is unclear how gut-derived signals modulate macrophage polarization within the EMS microenvironment.

In this study, we establish mouse models of EMS and subsequently apply human fecal microbiota transplantation (FMT) and antibiotic treatment to modulate gut microbiota composition. Pg is identified as a key microbial species that attenuates EMS progression. We show that Pg reshapes bile acid metabolism, leading to elevated 7-KLCA levels. Cellular studies demonstrate that 7-KLCA acts via Takeda G protein-coupled receptor 5 (TGR5, also known as GPBAR1) to suppress PPARγ and activate GPR132, thereby promoting M1 macrophage polarization and enhancing efferocytosis. These findings uncover a gut-metabolite-immune regulatory circuit that links microbial activity to macrophage-mediated immunomodulation in EMS and suggest a potential microbiota-based therapeutic approach.

## Results

### Gut microbiota modulates EMS pathogenesis in dual murine models

**Fig. 1:**
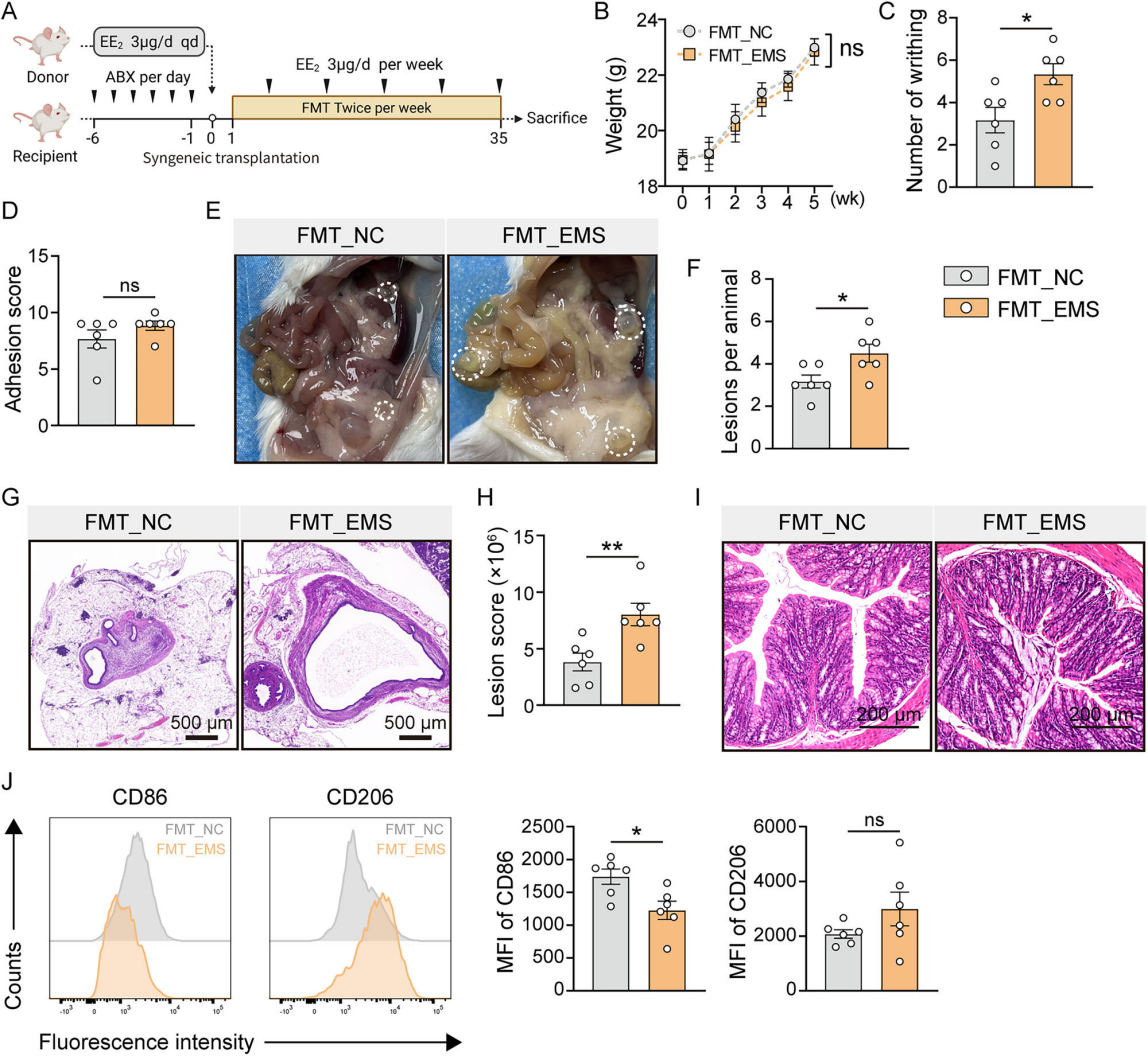
Fecal microbiota transplantation from EMS patients exacerbates endometriosis in mice. **A** Schematic illustration of the animal experiments. After one week of concurrent EE_2_ injections and antibiotic cocktail treatment, mice received twice-weekly FMT either from EMS patients or from healthy controls with weekly EE2 injections. **B** Body weight changes (*n* = 6/group). **C** Number of writhing events (*n* = 6/group). **D** Adhesion scores (*n* = 6/group). **E** Representative images of intraperitoneal endometriotic lesions (white circles). **F** Lesion number per mouse (*n* = 6/group). **G** Representative images of ectopic lesions stained with H&E (magnification, 40×; scale bar = 500 μm). **H** Lesion histopathology scores (*n* = 6/group). **I** Representative images of intestinal tissues stained with H&E. (magnification, 200×; scale bar = 200 μm). **J** The MFI of CD86 and CD206 on peritoneal lavage-derived macrophages was measured by flow cytometry (*n* = 6/ group). Results were expressed as mean ± SEM. For **B**, two-way ANOVA followed by Bonferroni’s multiple-comparison test was performed for statistical analysis; for **C**, **F**, **H**, and **J**, data were analyzed using a two-tailed *t*-test; for panel **D**, the two-sided Mann–Whitney *U* test was performed for statistical analysis. *p < 0.05, **p < 0.01, ***p < 0.001. ABX antibiotic cocktail treatment, FMT fecal microbiota transplantation, NC normal control, EMS endometriosis, ns not significant, EE_2_ estradiol benzoate, H&E Hematoxylin & Eosin, MFI mean fluorescence intensity.

To investigate the role of the gut microbiota in EMS, we established a FMT model. Mice were randomly assigned to receive microbiota from either EMS patients (FMT_EMS) or normal controls (FMT_NC) (Fig. 1A). This procedure did not result in significant changes in body weight (Fig. 1B). Pain responses, assessed by oxytocin-induced writhing assays, revealed a significantly higher number of abdominal contractions in the FMT_EMS group compared to FMT_NC controls (Fig. 1C). While adhesion scores appeared numerically elevated in the FMT_EMS group (Fig. 1D), no significant difference was observed. In contrast, consistent with visual evidence of increased lesion number and size in the FMT_EMS group (Fig. 1E–G), lesion scores were significantly higher than those in the FMT_NC group (Fig. 1H). No overt structural alterations were observed in intestinal tissues between the two groups (Fig. 1I).

Given the proved role of macrophages in EMS pathogenesis—whereby early M1 polarization gives way to an immunosuppressive M2 phenotype promoting angiogenesis, adhesion, and suppressing immune cell-mediated lesion clearance^11,12^—we evaluated macrophage subsets by flow cytometry. Analysis of surface marker expression revealed a significant reduction in CD86 mean fluorescence intensity (MFI) in the FMT_EMS group, while CD206 expression showed no significant difference (Fig. 1J). Collectively, these findings suggest that EMS-associated gut microbiota may suppress M1 macrophage polarization, thereby contributing to disease progression.

**Fig. 2:**
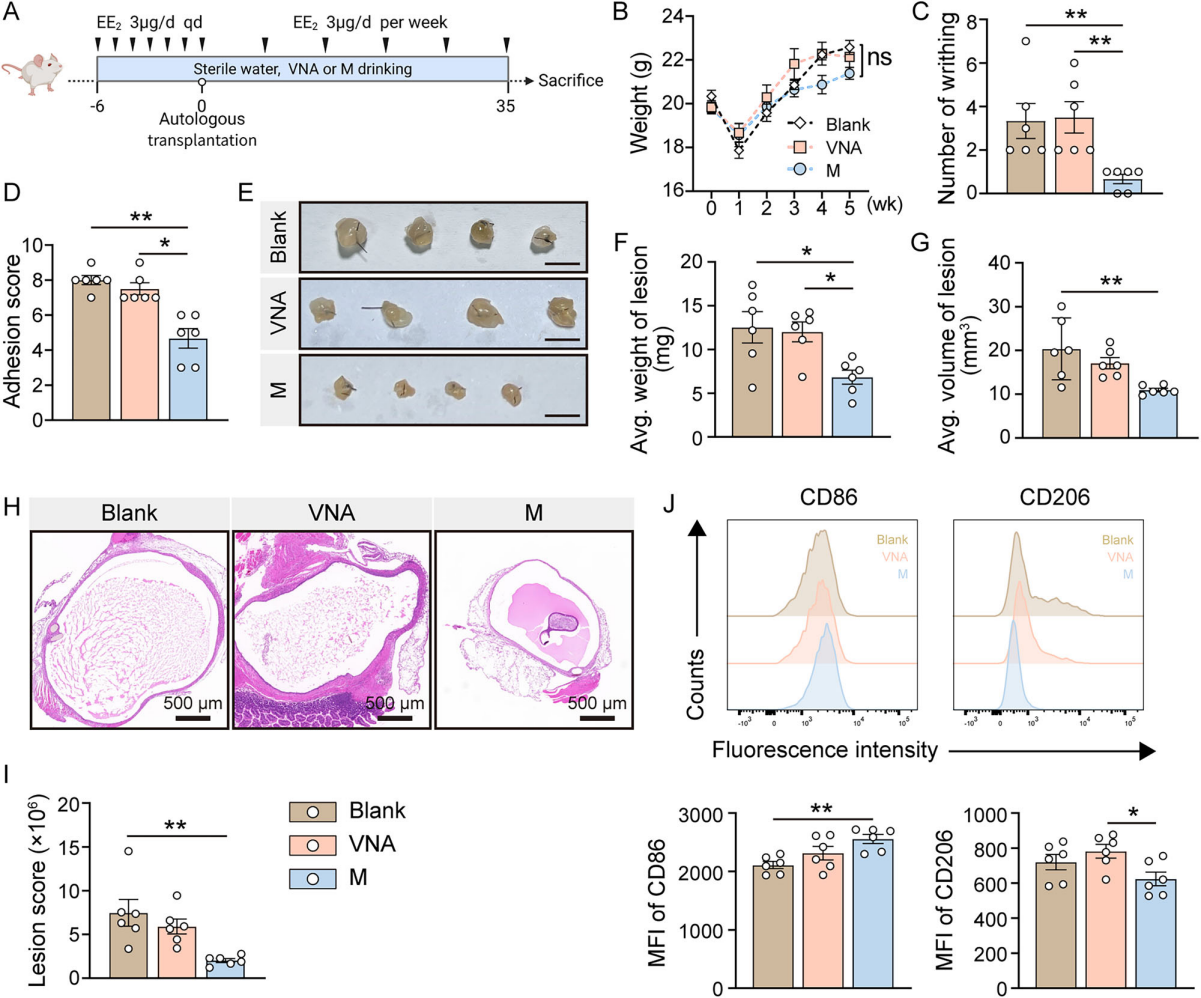
Antibiotic therapy with metronidazole slows EMS progression in mice. **A** Schematic illustration of the animal experiments. Mice were injected with EE_2_ for 7 days, followed by weekly injections. The mice were then randomly assigned to three groups. Except for the control group, which received sterile water, the other groups received VNA or M drinking water respectively. **B** Body weight changes (*n* = 6/group). **C** The number of writhing (*n* = 6/group). **D** Adhesion scores (n = 6/group). **E** Ectopic endometriotic lesion representative images (scale bar = 5 mm). **F** The average weight of lesions (*n* = 6/group). **G** The average volume of lesions (*n* = 6/group). **H** H&E-stained ectopic lesions (magnification, 30×; scale bar = 500 μm). **I** Lesion score (*n* = 6/group). **J** The MFI of CD86 and CD206 was measured by flow cytometry in macrophages derived from peritoneal lavage (*n* = 6/group). Data was expressed as the mean ± SEM. In **B**, data were determined using the two-way ANOVA followed by Bonferroni’s post-hoc test; for **C,** **D**, data were analyzed using Kruskal–Wallis test followed by Dunn’s test; for **F**, **G**, **I**, **J**, the one-way ANOVA followed by Bonferroni’s post-hoc test was used for statistical analysis. *p < 0.05, **p < 0.01, ***p < 0.001. EE_2_ estradiol benzoate, EMS endometriosis, VNA vancomycin, neomycin and ampicillin, M metronidazole, ns not significant, MFI mean fluorescence intensity.

Building upon an autograft-derived endometriosis model (designed to avoid immune microenvironmental perturbations by syngeneic transplantation^19^), we developed a murine system with antibiotic-induced microbial perturbation to investigate the impact of gut microbial compositional changes on EMS pathogenesis (Fig. 2A). Three experimental groups were established: (i) the VNA group received vancomycin, neomycin, and ampicillin, targeting anaerobic depletion while sparing metronidazole-sensitive species; (ii) the M group was treated with metronidazole; and (iii) the Blank group received sterile water. Throughout the experimental period, no significant differences in body weight were observed among the groups (Fig. 2B). Notably, oxytocin-induced writhing was significantly reduced in the M group compared to both VNA and Blank groups (Fig. 2C). Phenotypic analysis demonstrated that metronidazole treatment markedly alleviated disease severity, as indicated by reduced adhesion scores (Fig. 2D), smaller lesion size (Fig. 2E), and decreased lesion weight and volume (Fig. 2F, G). Histological examination revealed thinner stromal and epithelial layers in lesions from the M group (Fig. 2H), consistent with lower lesion scores (Fig. 2I). Flow cytometry further showed a reduction in CD206⁺ M2 macrophages and a marginal increase in CD86⁺ M1 macrophages in the M group (Fig. 2J), suggesting that metronidazole-mediated microbial shifts may suppress M2 polarization. These findings align with the previous report^18^ and further support a model in which gut microbiota modulate EMS pathogenesis through macrophage phenotypic reprogramming.

### Pg mediates the therapeutic effects of metronidazole in an EMS model

**Fig. 3:**
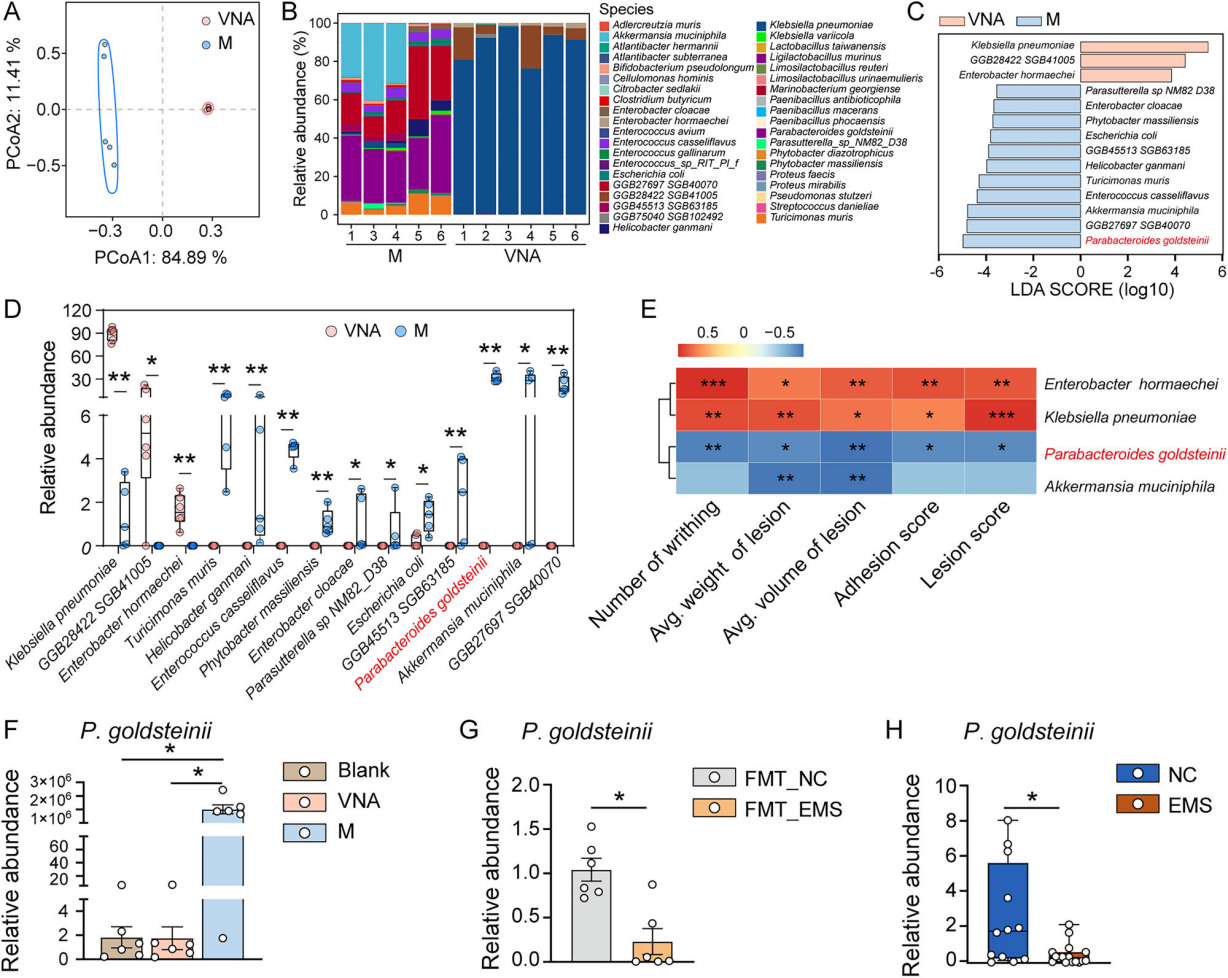
Metronidazole induced GM structural changes in EMS mice. **A** Principal coordinate analysis of β-diversity (based on the Bray-Curtis matrix) revealed significant separation between the M and VNA groups in species-level microbial community structure (M group *n* = 5, VNA group *n* = 6). **B** Overall microbial composition at the species level. **C** Linear discriminant analysis effect size (LEfSe) plots to identify the bacterial strains that characterize M versus VNA group. **D** Comparison of relative abundance of microbial species between M and VNA groups (M group *n* = 5, VNA group *n* = 6). Results were expressed as median and IQR. All plotted relative abundance values are non-zero measurements. **E** Correlation analysis between the relative abundance of microbial species and the observed phenotypes of mice. **F** Comparative analysis of Pg relative abundance across Blank, VNA, and M groups (*n* = 6/group). **G** The relative abundance of Pg between FMT_NC and FMT_EMS groups (*n* = 6/group). **H** The relative abundance of Pg between Non-EMS controls (*n* = 12) and EMS patients (*n* = 16). Data in **F**, **G** were presented as mean ± SEM. In **D**, **G** data were analyzed using the two-tailed *t*-test. In **E**, Spearman’s correlation test was used for statistical analysis. The differences in **F** between groups were compared using one-way ANOVA followed by Bonferroni’s post-hoc test for multiple comparisons. Data in panel **H** was presented as median and interquartile range (IQR) and was analyzed using Mann–Whitney *U* test. *p < 0.05, **p < 0.01, ***p < 0.001. FMT fecal microbiota transplantation, NC normal control, EMS endometriosis, VNA vancomycin, neomycin, and ampicillin, M metronidazole.

To elucidate microbiota-dependent mechanisms underlying metronidazole-mediated attenuation of EMS, we performed strain-resolved metagenomic sequencing on fecal samples from VNA- and M-treated mice. Principal coordinates analysis (PCoA) revealed that antibiotic perturbation led to substantial shifts in gut microbiota composition, with axis 1 and axis 2 explaining 84.89% and 11.41% of the total variance, respectively (Fig. 3A). Species-level taxonomic profiling demonstrated clear distinctions between the two treatment groups (Fig. 3B). LEfSe analysis (log₂ fold change > 3.5) further identified 14 differentially abundant taxa (Fig. 3C). Notably, the M group exhibited enrichment of putative probiotic species such as Pg and *Akkermansia muciniphila*, while opportunistic pathogens including *Klebsiella pneumoniae* and *Enterobacter hormaechei* were significantly depleted (Fig. 3D). Correlation analyses using Spearman’s rank correlation revealed that Pg and *Akkermansia muciniphila* were inversely associated with multiple clinical indices of EMS severity, including adhesion scores, frequency of abdominal writhing, lesion weight/volume, and histopathological scores. In contrast, *Klebsiella pneumoniae* and *Enterobacter hormaechei* showed positive correlations with these disease parameters (Fig. 3E). Among these taxa, only Pg showed a consistent increase in relative abundance in both metronidazole-treated mice and those colonized with microbiota from healthy controls, as confirmed by RT-qPCR (Fig. 3F, G and Supplementary Fig. 1A, B). Importantly, in line with these findings, we observed a significant reduction in the relative abundance of Pg in fecal samples from EMS patients compared to healthy controls (Fig. 3H). Collectively, these results suggest a potential role for Pg in modulating EMS progression.

**Fig. 4:**
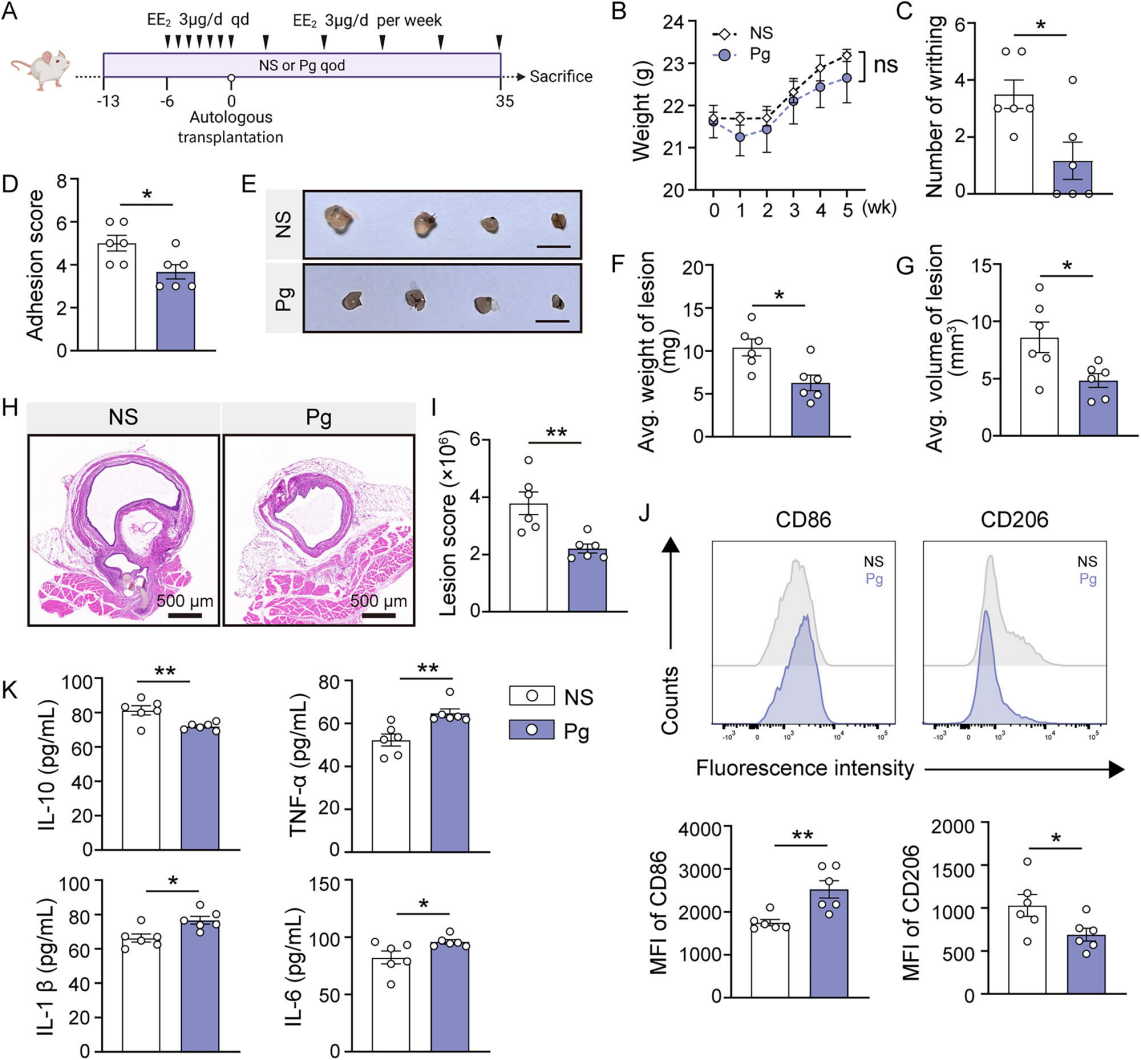
Pg attenuates EMS progression. **A** The schematic diagram of the animal experimental design. The mice were administered EE_2_ via daily injections for 7 days, followed by weekly injections thereafter. They were then divided into two groups and received NS or Pg respectively. **B** Body weight changes (*n* = 6/group). **C** The number of writhing (*n* = 6/group). **D** Adhesion scores (*n* = 6/group). **E** Ectopic endometriotic lesion representative images (scale bar = 5 mm). **F** The average weight of lesions (*n* = 6/group). **G** The average volume of lesions (*n* = 6/group). **H** Representative images of ectopic lesions from two groups stained with H&E (magnification, 30×; scale bar = 500 μm). **I** Lesion score (*n* = 6/group). **J** Peritoneal lavage-derived macrophages were analyzed for CD86 and CD206 MFI by flow cytometry (*n* = 6/group). **K** The serum levels of IL-10, TNF-α, IL-1β, and IL-6 were measured by ELISA (*n* = 6/group). Results were expressed as mean ± SEM. Two-way-ANOVA with Bonferroni post-hoc test was performed for data analysis in panel **B**. The two-sided Mann-Whitney U test was used to analyze TNF-α concentrations and CD86 MFI levels. All additional data was analyzed with a two-tailed *t*-test. *p < 0.05, **p < 0.01, ***p < 0.001. EE_2_ estradiol benzoate, Pg *Parabacteroides goldsteinii*, NS normal saline, ns not significant, EMS endometriosis, MFI mean fluorescence intensity.

To directly assess whether Pg contributes to the protective effects of metronidazole, mice were orally administered Pg prior to surgical induction of EMS (Fig. 4A). Consistent with previous findings, no significant differences in body weight gain were observed between groups (Fig. 4B). However, Pg-pretreated mice exhibited a significant reduction in oxytocin-induced writhing responses and abdominal adhesion scores (Fig. 4C, D). Gross pathological evaluation revealed visibly smaller lesions with significantly decreased lesion weight and volume (Fig. 4E–G), indicating a mitigated disease phenotype following Pg exposure. Histological examination confirmed that Pg-treated mice displayed thinning of both stromal and epithelial layers in endometriotic lesions, along with significantly lower lesion scores (Fig. 4H, I). Flow cytometry analysis further demonstrated a significant increase in CD86 MFI and a concurrent reduction in CD206 MFI in Pg-treated mice compared to saline controls (Fig. 4J), suggesting inhibition of M2 macrophage polarization. Consistently, serum cytokine analysis showed a reduction in IL-10 levels—an anti-inflammatory cytokine associated with M2 macrophages—accompanied by elevated levels of M1-associated proinflammatory cytokines, including TNF-α, IL-1β, and IL-6 (Fig. 4K). These findings provide compelling evidence that Pg contributes to the therapeutic efficacy of metronidazole in EMS by suppressing M2 macrophage polarization, thereby highlighting its potential as a microbial-based therapeutic candidate for endometriosis.

### Bile acid metabolism mediates Pg-driven amelioration of EMS

The gut microbiota produces a broad spectrum of bioactive metabolites that influence disease initiation and progression^20,21^. However, whether Pg, a prevalent gut commensal, modulates EMS phenotypes through its secreted metabolites remains unclear. To address this, we orally administered Pg culture medium (PgCM) to EMS mice and evaluated its therapeutic efficacy (Supplementary Fig. 2A). Unexpectedly, PgCM treatment alone failed to improve disease features, as indicated by unaltered body weight and lesion size, abdominal adhesion scores, writhing frequency, and lesion burden compared to positive control (Supplementary Fig. 2B–G). In addition, no significant differences in peritoneal macrophage polarization profiles were observed between the PgCM and control groups (Supplementary Fig. 2H). These findings suggest that direct metabolite exposure may be insufficient to replicate the therapeutic effects of live Pg and highlight the need for further investigation into the specific mediators involved.

**Fig. 5:**
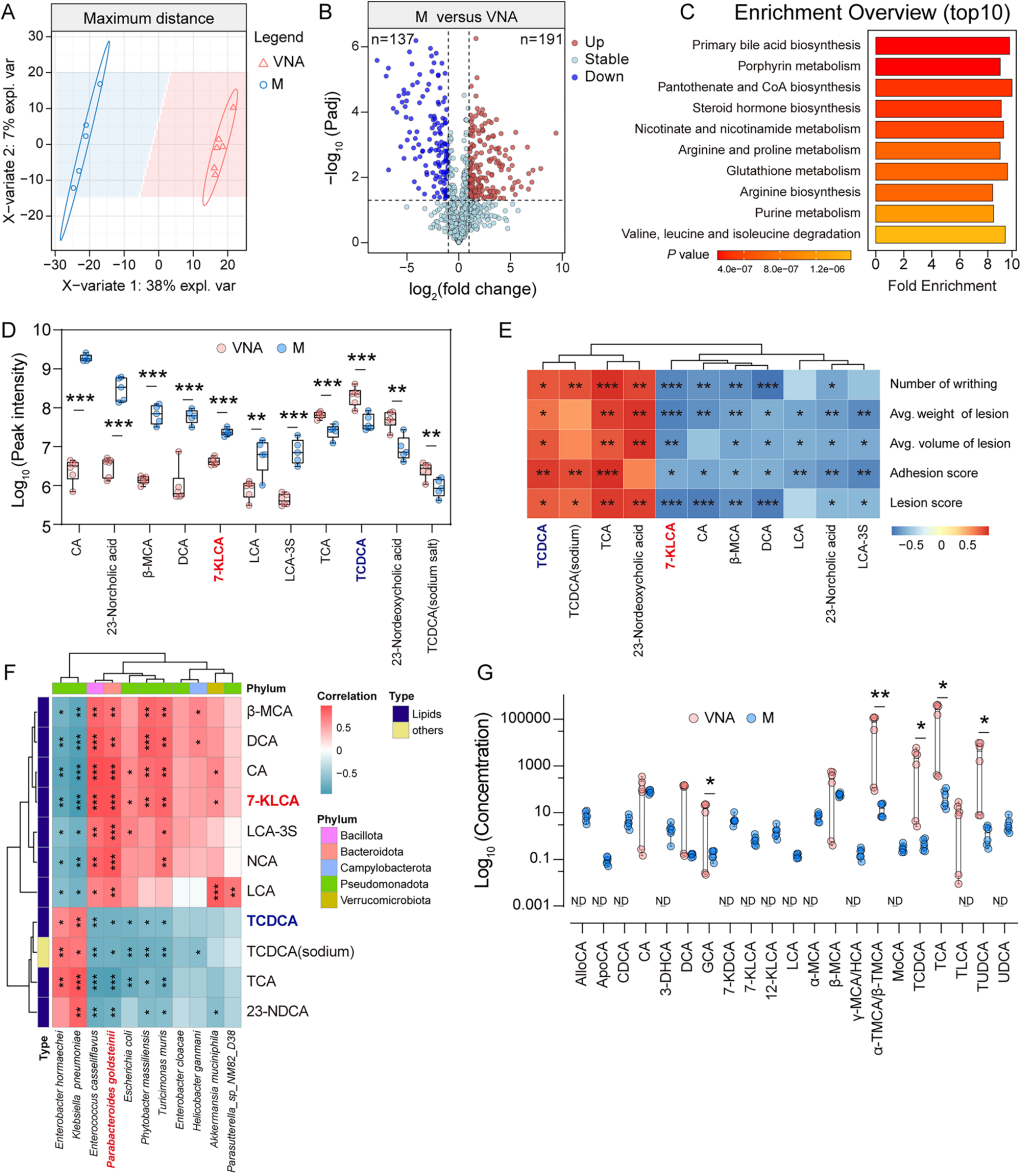
Metronidazole modulates bile acid metabolism. **A** Principal component analysis (PCA) score plot of cecal metabolomic (M group *n* = 5, VNA group *n* = 6). **B** Volcano plot illustrates the significant differences in metabolites enriched between M and VNA group. Each point represents one metabolite. **C** The top 10 most significantly enriched functional terms were identified based on *p*-value and enrichment ratio. **D** Peak intensity of the BAs (M group *n* = 5, VNA group *n* = 6). **E** Correlation analysis between the BAs and the observed phenotypes of mice. **F** Correlations between the relative abundance of microbial species and bile acids. **G** Targeted BA concentration in feces of VNA and M groups (*n* = 6/group). Concentrations are in nmol/g. Data was presented as median and IQR, analyzed by the two-tailed *t*-tests. In **E**, **F**, Spearman’s correlation test was used for statistical analysis. *p < 0.05, **p < 0.01, ***p < 0.001. VNA vancomycin, neomycin and ampicillin, M metronidazole, ND not detected.

To explore potential metabolite-based mechanisms, we performed untargeted metabolomic profiling of cecal contents from VNA- and M-treated mice. Principal component analysis revealed a clear separation between metabolic profiles of the two groups (Fig. 5A). In total, 191 metabolites were upregulated and 137 were downregulated in the M group (Fig. 5B). KEGG pathway enrichment analysis identified primary bile acid biosynthesis as significantly enriched in the M group (Fig. 5C). We next assessed fecal bile acid composition and observed that cholic acid (CA), β-muricholic acid (β-MCA), deoxycholic acid (DCA), 7-KLCA, and lithocholic acid (LCA) were significantly enriched in the M group, while taurocholic acid (TCA), taurochenodeoxycholic acid (TCDCA), and 23-nordeoxycholic acid were elevated in the VNA group (Fig. 5D). Furthermore, Spearman correlation analysis revealed strong associations between specific bile acids and key clinical metrics of EMS (Fig. 5E). Given that BA composition is dynamically shaped by gut microbiota^22^, we next analyzed the relationships between BA levels and bacterial species. Pearson’s correlation analysis indicated that the relative abundance of 11 bacterial taxa, including Pg, was closely associated with BA concentrations (Fig. 5F).

To identify the key bile acids driving these effects, we conducted targeted quantitative metabolomics. The results confirmed distinct bile acid clustering patterns, with glycocholic acid (GCA), 7-KLCA, and chenodeoxycholic acid (CDCA) elevated in the M group, whereas TCDCA, TCA, and tauroursodeoxycholic acid (TUDCA) were predominantly enriched in the VNA group (Fig. 5G). Collectively, these findings support a model in which Pg modulates bile acid metabolism, thereby contributing to the attenuation of EMS pathophysiology.

### Pg-mediated 7-KLCA alleviates EMS via the gut microbiota-immune Axis

As illustrated in Supplementary Fig. 3A, members of the *Parabacteroides* genus have previously been shown to express bile salt hydrolase (BSH) and 7α-hydroxysteroid dehydrogenase (7α-HSDH), key enzymes involved in the bioconversion of TCDCA to 7-KLCA^23,24^. To determine whether Pg possesses this metabolic capacity, we conducted targeted in vitro fermentation assays. As expected, supplementation of TCDCA to Pg cultures led to the accumulation of both 7-KLCA and its metabolic intermediate, CDCA (Supplementary Fig. 3B, C). Quantitative analyses revealed elevated CDCA/TCDCA and 7-KLCA/CDCA ratios in the anaerobic culture supernatants (Supplementary Fig. 3D), consistent with BSH-mediated deconjugation followed by 7α-HSDH-driven oxidation.

To further investigate microbial feedback interactions, we examined the effects of TCDCA and 7-KLCA on Pg growth in vitro. TCDCA at concentrations ≤200 μM did not significantly alter bacterial growth, whereas 500 μM TCDCA markedly suppressed Pg proliferation (Supplementary Fig. 3E). In contrast, 7-KLCA exerted no significant effects on Pg growth at any tested concentration (Supplementary Fig. 3F). To explore the in vivo relevance of this pathway, we administered Pg cultures supplemented with TCDCA (PgTCM) to EMS-induced mice via oral gavage over a 5-week period (Supplementary Fig. 2A). Notably, PgTCM-treated mice exhibited significant attenuation of EMS-related phenotypes—including reduced fibrotic adhesions, pelvic pain, and lesion progression—when compared with both Vehicle- and PgCM-treated groups (Supplementary Fig. 2B–G). Consistent with these phenotypic improvements, an increase in CD86 MFI was observed in peritoneal macrophages, whereas CD206 MFI remained unchanged (Supplementary Fig. 2H). These findings suggest that Pg may contribute to EMS amelioration through modulation of bile acid metabolism, particularly via 7-KLCA.

**Fig. 6:**
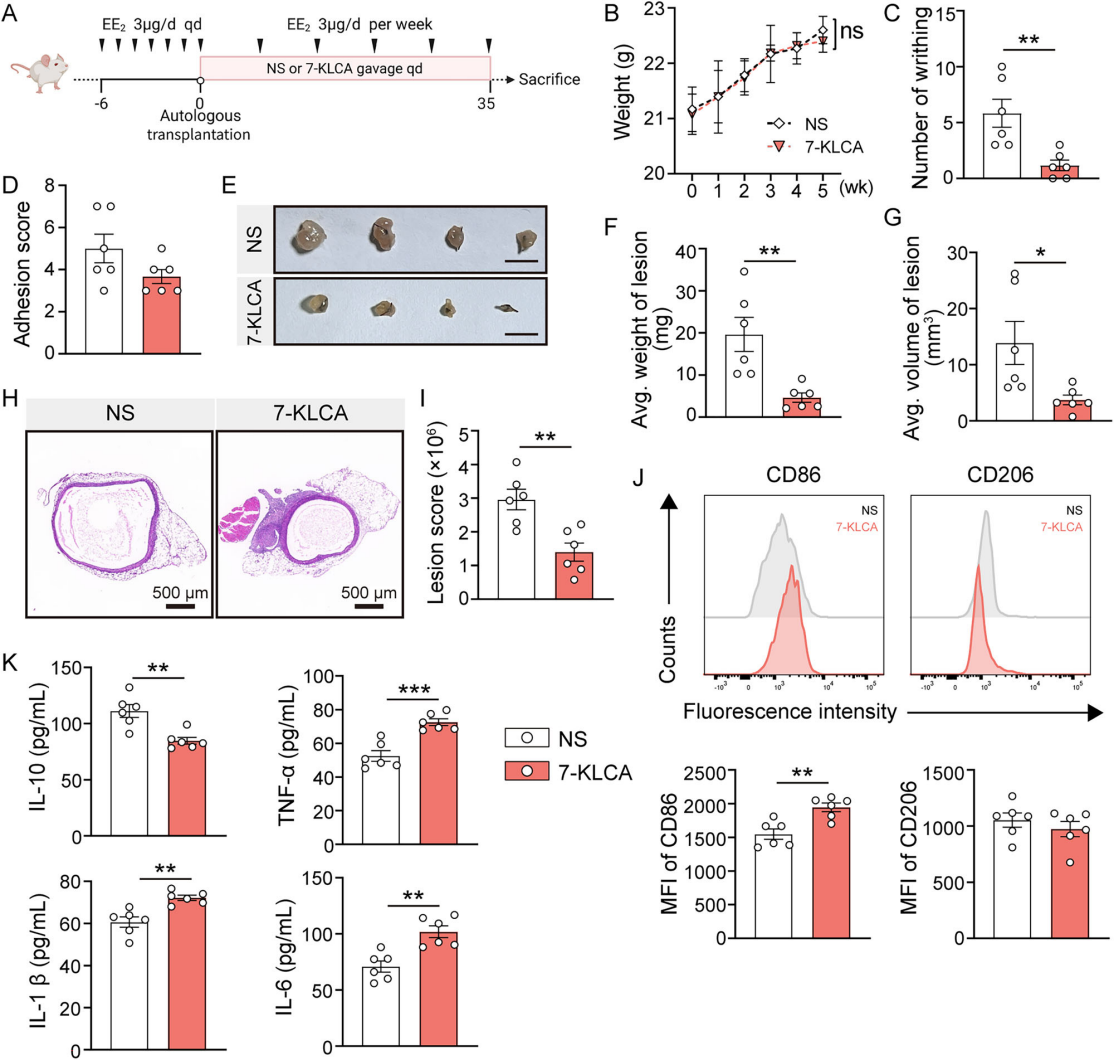
7-KLCA supplementation alleviates EMS progression. **A** Schematic representation of the animal experiment. Mice were subjected to 7-day daily EE_2_ injections followed by weekly maintenance, then stratified into two groups receiving either NS or 7-KLCA via oral gavage every day for five weeks. **B** Body weight changes (*n* = 6/group), as determined using a two-way ANOVA followed by Bonferroni’s post-hoc test. **C** The number of writhing (*n* = 6/group). **D** Adhesion scores (*n* = 6/group). **E** Ectopic endometriotic lesion representative images (scale bar = 5 mm). **F** The average weight of lesions (*n* = 6/group). **G** The average volume of lesions (*n* = 6/group). **H** Representative images of ectopic lesions from two groups stained with H&E (magnification, 30×; scale bar = 500 μm). **I** Lesion score (*n* = 6/group). **J** The MFI of CD86 and CD206 on peritoneal lavage-derived macrophages was measured by flow cytometry (*n* = 6/group). **K** The serum levels of IL-10, TNF-α, IL-1β, and IL-6 were detected using ELISA (*n* = 6/group). Results were expressed as mean ± SEM and analyzed using the two-tailed *t*-test. *p < 0.05, **p < 0.01, ***p < 0.001. EE_2_ estradiol benzoate, 7-KLCA 7-ketolithocholic acid, NS normal saline, ns not significant, EMS endometriosis, MFI mean fluorescence intensity.

To directly evaluate the therapeutic potential of Pg-derived 7-KLCA, mice were administered purified 7-KLCA following EMS induction via uterine auto-transplantation (Fig. 6A). Consistent with prior safety data, 7-KLCA treatment did not affect body weight (Fig. 6B). Behavioral analysis revealed a significant reduction in oxytocin-induced writhing, as well as decreased abdominal adhesion severity in the 7-KLCA group compared with normal control (Fig. 6C, D). Morphometric assessments confirmed a marked reduction in lesion volume and weight (Fig. 6E–G), and histopathological analysis revealed diminished epithelial and stromal thickening, along with reduced lesion severity scores (Fig. 6H, I).

As previously reported, restoration of the disrupted M1/M2 macrophage balance may facilitate immune clearance of ectopic endometrial tissue within the peritoneal cavity^6^. In support of this, flow cytometry showed a significant increase in CD86 MFI and a corresponding decrease in CD206 MFI in 7-KLCA-treated mice compared to NS control (Fig. 6J), indicating M1 polarization with concurrent suppression of the M2 phenotype. Serum cytokine profiling further corroborated this immune shift: M1-associated proinflammatory cytokines IL-1β, TNF-α, and IL-6 were elevated, whereas the M2-associated anti-inflammatory cytokine IL-10 was markedly reduced (Fig. 6K). These results demonstrate that 7-KLCA mitigates EMS pathogenesis by modulating the gut microbiota-immune axis. Together with the established role of Pg in producing 7-KLCA^24^, our findings suggest that Pg may confer benefits via this metabolite.

### 7-KLCA promotes M2-to-M1 phenotypic switch by targeting TGR5

**Fig. 7:**
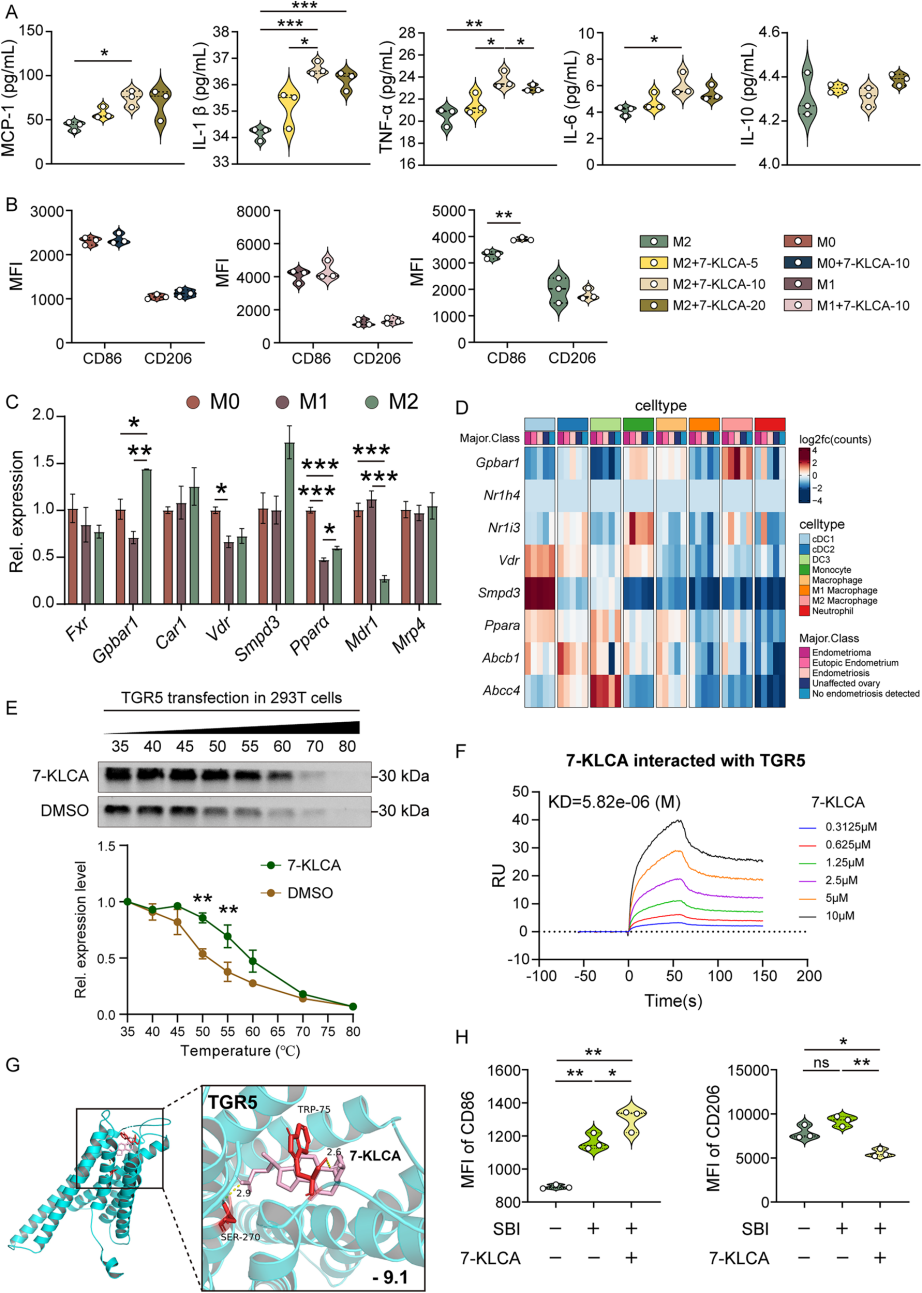
TGR5 promotes macrophage polarization from the M2 to M1 phenotype. **A** The MCP-1, IL-1β, TNF-α, IL-6, and IL-10 levels in M2 culture medium supernatant under 7-KLCA treatment at various concentrations (0 μM, 5 μM, 10 μM, and 20 μM) were measured by ELISA (*n* = 3/group). **B** Bone marrow-derived macrophages (BMDMs) were analyzed for the MFI of CD86 and CD206 in their M0, M1, and M2 phenotypes after treatment with 10 μM 7-KLCA for 24 h (*n* = 3/group). **C** Relative expression of bile acid-related receptors in M0, M1, and M2 macrophages by RT-qPCR. The data were presented as mean ± SEM. **D** The heat map shows the average expression of bile acid-related genes across different histopathological types and various immune cell types based on data set (GSE213216) analysis. **E** Comparative western blot analysis of TGR5 thermal stability in lysates of 293T cells overexpressing TGR5 treated with or without 10 µM 7-KLCA. **F** SPR analysis for TGR5 protein with different doses of 7-KLCA. **G** Molecular docking analysis of TGR5 and 7-KLCA, and the potential interaction binding site. TGR5 (light blue) and the ligand 7-KLCA (pink) bind via key amino acid residues TRP-75 and SER-270 (red), with their interaction distances measured at 2.6 and 2.9 Å and a calculated binding energy of -9.1 kcal/mol. **H** M2 macrophages were treated as indicated: Control (DMSO), SBI-115 (5 µM), or SBI-115 plus 7-KLCA (10 µM). Flow cytometric analysis of CD86 and CD206 expression (MFI) is shown. Data shown as median and IQR unless otherwise indicated. The differences between groups were compared using one-way ANOVA followed by Bonferroni’s post-hoc test for multiple comparisons in **A**, **B**, **C**, **H** (except for the Rel. expression of *Fxr*, which was analyzed by Kruskal–Wallis test followed by Dunn’s test). For Figure E, data were analyzed by two‑way ANOVA followed by Bonferroni’s post‑hoc test. *p < 0.05, **p < 0.01, ***p < 0.001. 7-KLCA 7-ketolithocholic acid, TGR5 G protein-coupled bile acid receptor 1.

To investigate the regulatory effects of 7-KLCA on macrophage polarization, BMDMs were polarized in vitro into M0, M1, and M2 phenotypes. Cells were then treated with 7-KLCA at concentrations ranging from 0 to 20 μM for 24 h, followed by cytokine quantification in cell supernatants using ELISA. In M2 macrophages, 7-KLCA induced a concentration-dependent increase in proinflammatory cytokines, including MCP-1, IL-1β, TNF-α, and IL-6, with the maximal effect observed at 10 μM, while IL-10 levels remained unchanged (Fig. 7A). These cytokine changes were not observed in M0 or M1 macrophages (Supplementary Fig. 4A, B). Consistently, flow cytometry analysis revealed a significant upregulation of CD86 MFI specifically in M2 macrophages following 7-KLCA treatment, with no comparable effect in M0 or M1 subsets (Fig. 7B). Together, these data suggest that 7-KLCA selectively promotes the phenotypic conversion of M2 macrophages toward a proinflammatory M1-like state.

Given that bile acids typically exert their biological functions through BA receptors^25,26^, we next assessed the expression of several canonical BA receptors in macrophage subtypes. Analysis revealed that TGR5 (encoded by *Gpbar1*) expression was markedly enriched in M2 macrophages relative to M0 and M1 cells, while the expression of *Fxr*, *Car1*, *Vdr*, *Smpd3*, and *Mrp4* showed no significant variation across subtypes (Fig. 7C). To further support these findings, we analyzed a publicly available transcriptomic dataset (GSE213216), which demonstrated elevated GPBAR1 transcript levels in M2 macrophages within endometriosis lesions (Fig. 7D and Supplementary Fig. 5A,B).

To validate TGR5 as a functional receptor mediating 7-KLCA signaling, we performed a CETSA. Results showed that 7-KLCA increased the thermal stability of TGR5 in heat-denatured 293T cells overexpressing TGR5, indicating a direct ligand-receptor interaction (Fig. 7E). This was further confirmed by SPR analysis, which demonstrated dose-dependent binding of 7-KLCA to recombinant TGR5, with an equilibrium dissociation constant (*K*_D_) of 5.82 μM (Fig. 7F). To identify specific interaction sites between 7-KLCA and TGR5, we conducted molecular docking using the human TGR5 ligand-binding domain structure (PDB: 7BW0). The docking simulation suggested that 7-KLCA fits into the receptor’s ligand-binding pocket and forms predicted interactions with residues TRP-75 and SER-270 (Fig. 7G).

To further characterize the functional interaction between 7-KLCA and TGR5, we employed the selective TGR5 antagonist SBI-115. Treatment of M2 macrophages with SBI-115 alone significantly increased CD86 MFI, mirroring the effect of 7-KLCA. Co-treatment with 7-KLCA and SBI-115 resulted in a combined effect, leading to a greater elevation of CD86 and a more pronounced reduction in CD206 MFI than treatment with SBI-115 alone (Fig. 7H). This additive pharmacological profile indicates that 7-KLCA enhances the functional outcome of TGR5 antagonism, suggesting that 7-KLCA may function as an inhibitor of TGR5 signaling in macrophages.

### 7-KLCA augments macrophage efferocytosis by modulating the PPARγ-GPR132 axis

**Fig. 8:**
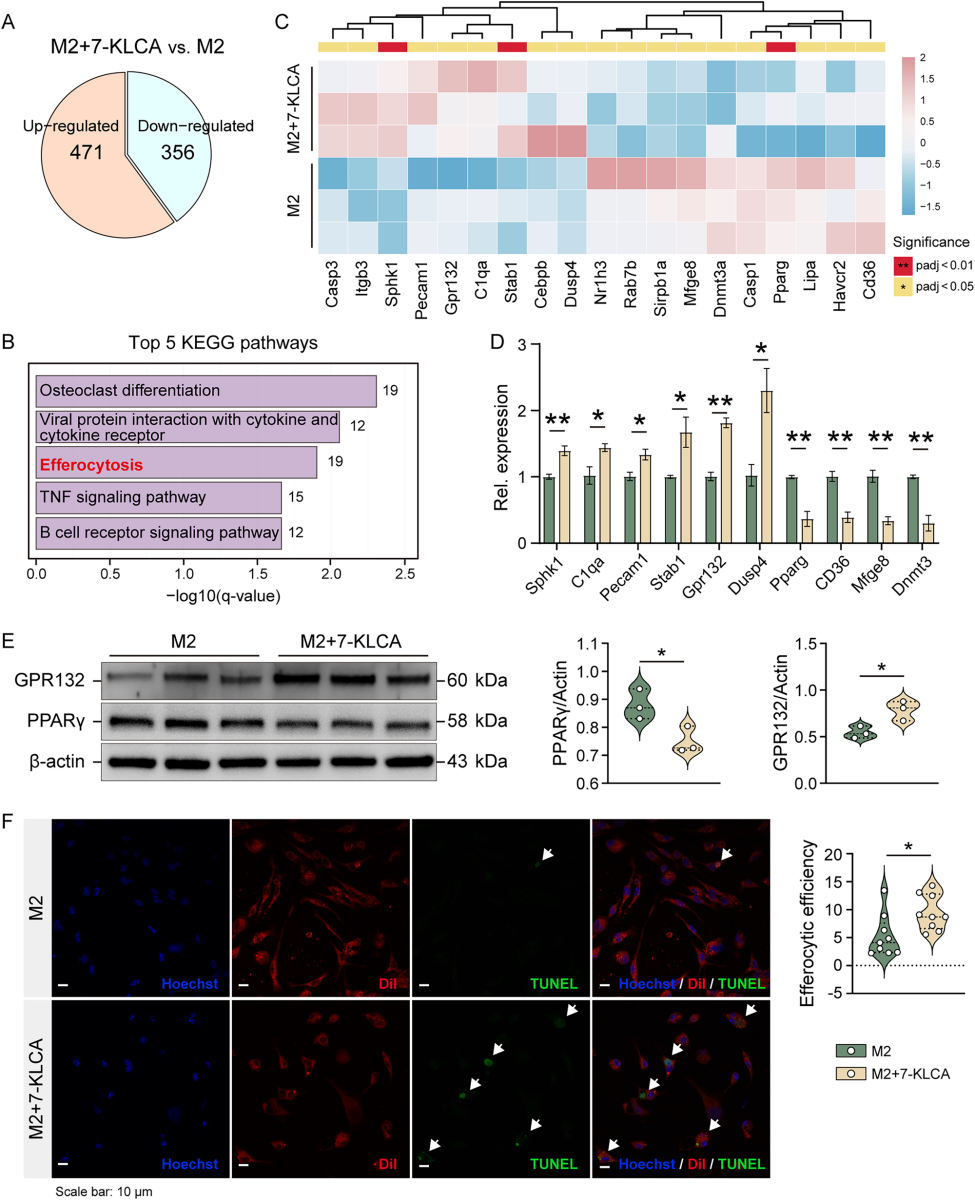
7-KLCA promotes macrophage efferocytosis by reprogramming a pro-efferocytic gene network. **A** Number of DEGs in M2 + 7-KLCA compared to M2, with padj < 0.05 (*n* = 3 /group). **B** Top 5 KEGG enrichment analysis regarding the DEGs of M2 vs. M2 + 7KLCA-10 (padj < 0.05). **C** DEGs of efferocytosis pathway between M2 and M2 + 7-KLCA (padj < 0.05, with padj < 0.01 highlighted in red). **D** Relative expression of efferocytosis related DEGs in each group. Data were expressed as mean ± SEM. **E** Representative western blot images and quantitative analysis of PPARγ and GPR132 levels in each group. Data shown as median and IQR. **F** Efferocytosis assay in M2 macrophages treated with or without 7-KLCA. (Left) Representative fluorescence microscopy images from three independent experiments, demonstrating efferocytosis activity in BMDMs treated with or without 7-KLCA (magnification, 630×; scale bar = 10 μm). For each experiment, three random fields per condition were captured. Dil-labeled BMDM were incubated with ACs labeled by TUNEL for 3 h. (Right) Quantification of efferocytic efficiency, presented as the percentage of macrophages that engulfed one or more ACs. The median efferocytosis rate was 4.08% (IQR: 2.33–7.60%) in the control group and 8.70% (IQR: 6.62–12.77%) in the 7-KLCA-treated group (Mann–Whitney U test). Results were analyzed using the two-tailed *t*-test in **D**, **E** (except for the Rel. expression of *Gpr132*, which was analyzed by Mann-Whitney U test). *p < 0.05, **p < 0.01, ***p < 0.001. ACs apoptosis cells.

To elucidate the molecular mechanisms underlying 7-KLCA-mediated modulation of macrophage function, we performed transcriptomic analysis on M2 macrophages treated with or without 10 μM 7-KLCA. RNA sequencing identified 417 upregulated and 356 downregulated DEGs (Fig. 8A). KEGG enrichment analysis revealed that these DEGs were predominantly associated with osteoclast differentiation, viral protein interaction and efferocytosis, alongside other pathways such as TNF signaling, and B cell receptor signaling pathways (Fig. 8B). Within the efferocytosis pathway, 19 genes were altered, with a heatmap highlighting the upregulation of *Sphk1* and *Stab1* and the downregulation of *Pparg* (Fig. 8C). To validate these changes, we selected 10 efferocytosis-related genes for quantitative PCR based on prior literature^27–34^. Expression of *Sphk1*, *C1qa*, *Pecam1*, *Stab1*, *Gpr132*, and *Dusp4* was significantly increased, whereas *Pparg*, *Cd36*, *Mfge8*, and *Dnmt3a* were notably downregulated (Fig. 8D). At the protein level, western blot confirmed that 7‑KLCA treatment suppressed PPARγ and concurrently upregulated GPR132 (Fig. 8E). Functionally, this molecular reprogramming was associated with enhanced efferocytic capacity, as the median efferocytic efficiency increased from 4.08% (IQR: 2.33–7.60%) in controls to 8.70% (IQR: 6.62–12.77%) in 7-KLCA-treated macrophages (*p* < 0.05, Mann–Whitney U test) (Fig. 8F).

Notably, PPARγ has been reported to regulate both macrophage differentiation^35^ and efferocytosis^36^, and it transcriptionally represses *Gpr132*—a validated promoter of efferocytosis in immune cells^37^. Our findings that 7-KLCA downregulates PPARγ and upregulates GPR132, alongside other pro-efferocytic genes (e.g., *Stab1*, *C1qa*), provide a mechanistic basis for the observed enhancement in efferocytic function^30,38^. Together, our findings suggest that 7-KLCA enhances macrophage efferocytosis through a mechanism that may involve a PPARγ-independent component, potentially mediated via the upregulation of GPR132 and other pro-efferocytic genes, thereby reshaping macrophage functional differentiation in the context of EMS.

## Discussion

Endometriosis (EMS) is a complex and heterogeneous disease influenced by hormonal, immune, and microbial factors^39,40^. Recent advances in EMS research have enabled comprehensive microbiome profiling across multiple anatomical sites, revealing spatially distinct microbial dysbiosis patterns in the gut, cervical mucus, and peritoneal fluid that are different from healthy controls^13,41,42^. Notably, the GM has emerged as a particularly promising diagnostic and therapeutic target^13^. Despite associations between gut dysbiosis and EMS, the causal pathways through which specific species or its derivative modulate the disease progression remain undefined. In this study, we identified Pg as a probiotic and its downstream metabolite 7-KLCA as a therapeutic metabolite, through microbiota manipulation and reshaping in murine EMS models as well as multi-omics integration.

Our findings provide direct evidence linking the gut microbiota to EMS pathology. Consistent with previous observations^18^, metronidazole treatment significantly attenuated EMS phenotypes. Based on metagenomic sequencing, we observed increased abundance of Pg and *Akkermansia muciniphila* in metronidazole-treated mice, both of which were negatively correlated with disease severity. Indeed, Pg is known for its probiotic potential; it produces key metabolites like acetate and butyrate, contributing to enhanced gut barrier function and improved glucolipid metabolism^24,43,44^. Immunomodulatory effects have also been reported, including the inhibition of the NF-κB pathway and the promotion of Treg differentiation^45,46^. Although studies report that butyrate (a key short chain fatty acid) improves EMS pathogenesis—and *Akkermansia muciniphila* as a major microbial source may contribute to this effect—no significant alterations in butyrate levels were detected in our untargeted metabolomics data^47–50^. Our integrated multi-omics analysis revealed that the therapeutic condition (metronidazole treatment), which enriched for Pg, was associated with a distinct reshaping of BA metabolism. Notably, Pg encodes BSH and 7α-HSDH, which convert TCDCA to 7-KLCA—a metabolite found to be functionally involved in disease modulation^24^. The stark contrast between the therapeutic effect of metronidazole and the lack of efficacy with broad-spectrum VNA treatment underscores that not all microbiota alterations are beneficial. Together, these findings suggest that metronidazole’s therapeutic effects may be mediated, at least in part, by specific, Pg-driven changes in bile acid metabolic outputs. Beyond Pg, other gut commensals with bile salt hydrolase (BSH) activity—such as various Bacteroides species which deconjugate primary bile acids—may likewise participate in shaping the host bile acid pool and its immunomodulatory potential^51^. Whether these BSH-active bacteria contribute to EMS pathogenesis through similar or distinct mechanisms remains an open question for future study. Furthermore, the immunomodulatory potential of *Akkermansia muciniphila* warrants further investigation to elucidate its complementary or synergistic role in EMS improvement.

The gut microbiota’s dynamic immunoregulatory network not only governs host immune homeostasis but also harbors untapped therapeutic potential^52,53^. Among the immune cell types influenced by microbial signals, macrophages are central players^54,55^. These highly plastic cells exist along a continuum from classically activated (M1) to alternatively activated (M2) states. In EMS, M2-polarized macrophages dominate the peritoneal cavity and ectopic lesions, facilitating immune suppression, neovascularization, and fibrotic remodeling^6,9^. Our results demonstrate that both Pg and its metabolite 7-KLCA can reprogram macrophage polarization in favor of an M1 phenotype, as evidenced by increased CD86 and decreased CD206 expression. This shift was corroborated by serum cytokine profiles showing upregulation of IL-1β, TNF-α, and IL-6, alongside reduced IL-10. Mechanistically, reduced M2 polarization may disrupt IL-10- and TGF-β-mediated angiogenesis and suppress neurogenesis, thereby alleviating pelvic pain and limiting lesion expansion^6,56^. These findings place macrophage plasticity at the core of gut-immune communication in EMS and identify it as a key therapeutic target.

Recent studies have further demonstrated that gut-derived microbial metabolites can activate intestinal immune cells and promote their migration to peripheral tissues, influencing disease outcomes at extraintestinal sites^57,58^. We show that 7-KLCA treatment of M2 macrophages significantly upregulated GPR132, a receptor that promotes M1 polarization and, as a scavenger receptor for apoptotic cells, facilitates chemotaxis to inflammatory lesions^31^. Our data support this paradigm and suggest that macrophages may serve as immunological intermediaries linking microbial metabolism to EMS pathology. However, whether intestinal macrophages directly migrate to the peritoneal cavity or whether circulating monocytes adopt a reprogrammed phenotype in response to systemic metabolites remains an open question requiring further investigation.

In addition to modulating polarization, 7-KLCA was found to enhance efferocytosis—a process by which macrophages clear apoptotic cells and debris to resolve inflammation^59^. Dysfunctional efferocytosis has been implicated in EMS, where the failure to remove cellular debris contributes to lesion implantation and persistence^60^. Transcriptomic profiling revealed that 7-KLCA treatment significantly enriched efferocytosis-related pathways and altered the expression of key regulators. Notably, it suppressed *Pparg* while upregulating pro-efferocytic genes, including *Gpr132*, *Sphk1*, *Stab1*, and *C1qa*^33,36,37,61^. These coordinated transcriptional changes were directly associated with a significant gain in efferocytic capacity. Our findings thus suggest that 7-KLCA promotes immune resolution not only by repolarizing macrophages but also by reprogramming a network of efferocytosis-related genes, with PPARγ suppression being a central but not necessarily exclusive event in this process.

An intriguing aspect of our findings is that 7-KLCA promotes a proinflammatory M1 macrophage shift via TGR5, a receptor often linked to anti-inflammatory and metabolic homeostasis. This apparent paradox may be explained by the context-dependent nature of bile acid signaling. Our pharmacological data using the TGR5 antagonist SBI-115 are consistent with a model in which 7-KLCA may act as an inhibitor or biased ligand of TGR5 in macrophages. While this inverse correlation invites speculation that TGR5 signaling may influence PPARγ activity—a key transcriptional driver of the M2 phenotype and efferocytosis—the exact nature of this interaction and its contribution to the observed pro-efferocytic effect require further genetic validation.

Despite these insights, our study has limitations. A comprehensive understanding of 7-KLCA’s in vivo behavior will be crucial for its translational development. Future studies utilizing labeled tracers are warranted to fully characterize its pharmacokinetics, biodistribution, and cellular uptake, which will provide a foundation for optimizing its therapeutic application. Furthermore, while the therapeutic effects of purified 7-KLCA demonstrate its sufficiency, an important future direction will be to establish its necessity within the Pg-mediated response. This could be achieved through studies employing Pg mutants genetically engineered to be deficient in 7-KLCA synthesis, which would further solidify the causal link and open avenues for engineering next-generation probiotics. Moreover, while our analysis focused on macrophage responses, the EMS immune microenvironment includes a broader array of immune cell types whose interactions and functions remain unexplored. Lastly, although we identified TGR5 as a molecular target of 7-KLCA, the downstream signaling events remain to be fully elucidated. While our data show an association between 7-KLCA treatment and reduced PPARγ expression, the precise role of TGR5 activation in this process and the extent to which the pro-efferocytic effects are PPARγ-independent require further dissection through genetic and rescue approaches. Moreover, definitive characterization of the mode by which 7-KLCA engages TGR5—whether through direct antagonism, biased signaling, or allosteric modulation—will require direct assessment of canonical downstream effectors in future studies.

**Fig. 9:**
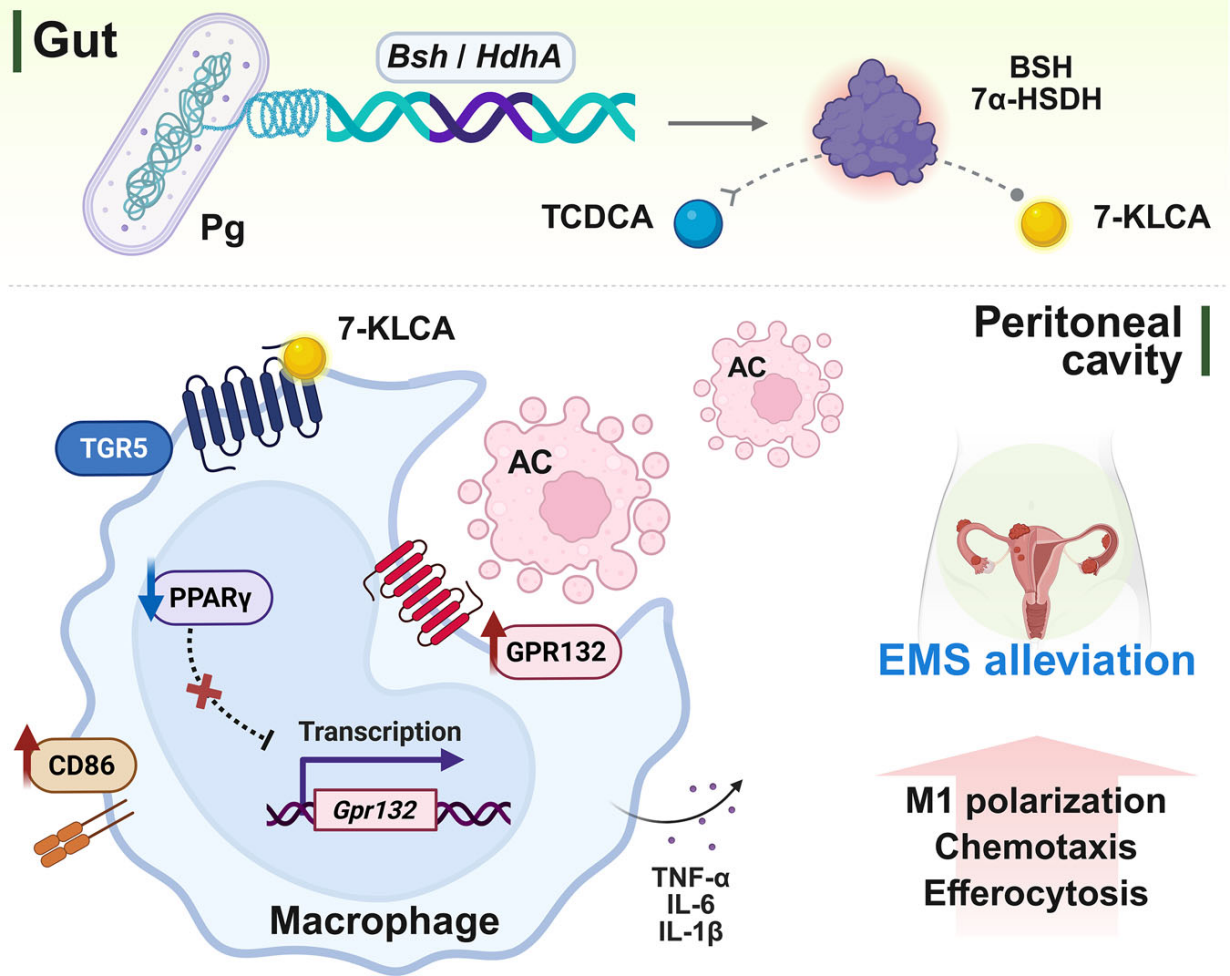
Schematic overview of the gut-immune axis in endometriosis suppression. Pg remodels bile acid metabolism to generate the microbially derived metabolite 7-KLCA. This metabolite targets macrophage TGR5 signaling, suppresses PPARγ expression, and thereby upregulates GPR132, thus promoting M1 polarization while enhancing efferocytosis. These immunomodulatory actions collectively mitigate endometriosis progression, establishing a mechanistic framework for microbiota-based therapeutic strategies. AC apoptosis cell.

In summary, our study uncovers a previously unappreciated gut-immune axis wherein Pg mitigates EMS progression via bile acid metabolic remodeling. The microbially derived metabolite 7-KLCA targets TGR5 signaling in macrophages, triggering a transcriptional reprogramming that includes the suppression of PPARγ. This, in turn, may alleviate the repression of downstream effectors like GPR132, contributing to their upregulation. This shift promotes a proinflammatory M1-like phenotype and enhances efferocytic capacity, collectively restraining endometriosis progression (Fig. 9). Our data further suggest that the enhancement of efferocytosis may involve both PPARγ-dependent and parallel pathways, revealing a new layer of complexity in microbial metabolite-mediated immunomodulation. These findings establish a mechanistic framework through which specific gut microbes and their metabolites can regulate peripheral immune function and modulate chronic gynecologic disease. Collectively, this work provides a strong rationale for microbiota-based therapeutic strategies targeting host-microbiota interactions in endometriosis.

## Methods

### Ethics statement and study participants

This study involving human participants was conducted in accordance with the International Ethical Guidelines for Research Involving Human Subjects and the Declaration of Helsinki. The protocol was reviewed and approved by the Medical Ethics Committee of Guangzhou Women and Children’s Medical Center, Guangzhou Medical University (approval number: 023A01). All participants provided written informed consent prior to enrollment, which detailed the study aims, procedures, and their right to withdraw at any time without penalty.

Women with a confirmed pathological diagnosis of EMS were recruited from the Department of Gynecology at Guangzhou Women and Children’s Medical Center, affiliated with Guangzhou Medical University. Age-matched women without EMS, confirmed by clinical and imaging evaluation, were enrolled as controls. Detailed demographic and clinical characteristics of all participants are provided in Supplementary Tables 1 and 3. Participants who had received antibiotics or probiotics within one month prior to sample collection were excluded. Additionally, individuals with any known conditions that could potentially affect gut microbiota composition—such as inflammatory bowel disease, irritable bowel syndrome, autoimmune disorders, or a history of major gastrointestinal surgery, among others—were also excluded. Fecal samples were aliquoted and stored at −80 °C until further use.

### Bacteria culture

The Pg strain ATCC BAA-1180 was cultured in modified GAM medium under strictly anaerobic conditions (90% N₂, 5% H₂, 5% CO₂) at 37 °C. Colony-forming units (CFUs) were determined by performing serial dilutions and plating on anaerobic blood agar plates, followed by incubation under the same anaerobic conditions. To collect bacterial supernatants, cultures were centrifuged sequentially at 3000 × *g* and 10,000 × *g* for 10 min each. The supernatants were filtered through a 0.22 μm pore-size filter to remove residual bacteria and then stored at −80 °C for further analysis.

The growth curve of Pg was assessed by measuring the OD600 values at 0, 3, 6, 9, 12, 18, 24, 30, and 36 h using a microplate reader after treatment with TCDCA and 7-KLCA at different concentrations (0 μM, 100 μM, 200 μM, and 500 μM).

To assess Pg-dependent metabolism of TCDCA, cultures were set up in medium alone or in medium supplemented with 200 µM TCDCA. A vehicle control containing 200 µM TCDCA in medium without Pg was included to account for any non-bacterial changes. After 24 h, supernatants were collected by sequential centrifugation and 0.22-μm filtration, aliquoted, and stored at −80 °C for subsequent analysis.

### Animal experiments

All animal experiments were conducted in accordance with the National Institutes of Health Guide for the Care and Use of Laboratory Animals, and protocols were approved by the Animal Care and Use Committee of Ruiye Bio-Tech Guangzhou Co., Ltd (RYEth-20250506699). Female BALB/c mice (8 weeks old) were purchased from GemPharmatech Co., Ltd. Mice were housed in a specific pathogen-free facility under a 12 h light/dark cycle, with ad libitum access to food and water.

For the syngeneic EMS model, estrous-stage donor mice received subcutaneous injections of estradiol benzoate (3 μg/mouse; HY-B1192, MCE, USA) daily for seven consecutive days. Uteri were excised, minced into uniform fragments in sterile phosphate-buffered saline (PBS), and intraperitoneally inoculated into recipient mice (two recipients per donor) using 16 G needles. To promote lesions development, recipient mice were administered weekly subcutaneous injections of estradiol benzoate throughout the experimental period. Mice were sacrificed at five weeks after modeling, and ectopic lesions located on the peritoneal wall, pancreas, intestinal mesentery, and peri-ovarian adipose tissue were counted, measured with a vernier caliper, and weighed using a microbalance. For the autologous model, female mice received estradiol benzoate for seven days to synchronize estrous cycles. Mice were then anesthetized with tribromoethanol (2.5% [w/v], 250 mg/kg body weight), and the left uterine horn was excised after ligation at the utero-tubal and utero-cervical junctions. Four uniform fragments (~2 mm³ each) were prepared using a biopsy punch and grafted onto the peritoneal wall of the same mouse. Mice were injected with estradiol benzoate weekly for five weeks. At endpoint, ectopic tissues on the peritoneal surface were dissected, measured, and weighed.

FMT was performed following a previously described protocol^62^. Briefly, mice were pretreated with an antibiotic cocktail consisting of vancomycin (100 mg/kg), neomycin sulfate (200 mg/kg), metronidazole (200 mg/kg), and ampicillin (200 mg/kg) via oral gavage once daily for six consecutive days. Endometriosis was then induced by syngeneic transplantation. One day post-modeling, mice were randomly assigned to two groups to receive fecal microbiota pellets from 5 EMS patients or 5 healthy women, respectively. Fecal samples from donors within each group were pooled to create a single inoculum per group. Fecal suspensions were administered twice per week until the end of the experiment.

For antibiotic intervention, mice received drinking water supplemented with either mixtures of vancomycin (0.5 g/L), neomycin (1 g/L), ampicillin (1 g/L), or metronidazole (1 g/L), starting six days prior to autologous transplantation and continuing throughout the study period. Control mice received sterile water.

After EMS induction, mice were administered 200 μL of Pg (5 × 10⁸ CFUs/mL) or saline every other day for five weeks. In parallel experiments, Pg was cultured in modified GAM medium with or without taurochenodeoxycholic acid (TCDCA, 200 μM). After 24 h, culture supernatants (Pg-conditioned medium [PgCM] or Pg-conditioned medium with TCDCA [PgTCM]) were collected and orally administered to mice daily for five weeks, starting one day post-surgery. Control mice received equal volumes of the culture vehicle (GAM medium).

For bile acid intervention, 7-KLCA (HY-W018512, MCE, USA) was first dissolved in dimethyl sulfoxide (DMSO) and then diluted in saline. Mice were orally gavaged with 200 μL of 7-KLCA (50 mg/kg/day) or saline alone daily for five weeks, followed by EMS induction.

Writhing behavior was induced by intraperitoneal injection of oxytocin (20 IU/kg; IO1340, Solarbio, China) on day 28 after EMS induction. Mice were observed for 30 min, and the number of writhes was recorded.

At endpoint, mice were deeply anesthetized with tribromoethanol (250 mg/kg, i.p.) and euthanized by cervical dislocation. Blood samples were collected and centrifuged at 3000 × *g* for 15 min at 4 °C to isolate serum. Peritoneal cells were harvested by injecting 5 mL of ice-cold saline into the cavity, followed by gentle shaking and collection. Organs were either snap-frozen and stored at −80 °C or fixed in 4% (v/v) paraformaldehyde and embedded in paraffin using standard histological protocols.

### Evaluation of adhesions

Immediately following the laparotomy and prior to any organ dissection for sample collection, macroscopic intra-abdominal adhesions were systematically evaluated by blinded observers. The scoring was performed using a previously reported system^63^, which quantifies adhesions based on three parameters: the extent of the adhesion area (scored 0–4), the type of adhesion tissue (scored 0–4), and its tenacity (scored 0–3). The scores from these categories were summed to yield a total adhesion score ranging from 0 (no adhesions) to 11 (most severe adhesions).

### Shotgun metagenomic analysis

Fresh fecal pellets were collected by placing individual mice in sterile, empty cages without bedding for 30 min. Genomic DNA was extracted and purified using the Stool DNA Extraction Mini Kit (DNS362-03, Mabio, China), following the manufacturer’s instructions. DNA quality and concentration were assessed, and qualified samples were used for metagenomic library construction. Sequencing was performed on the Illumina NovaSeq 6000 platform with 150 bp paired-end reads (PE150). Raw reads were quality-controlled using Fastp (v0.23.4) to remove adapter sequences, low-quality reads, and ambiguous bases, yielding clean data for downstream analysis. High-quality reads were de novo assembled into contigs (≥500 bp) using MEGAHIT (v1.2.9). Taxonomic profiling of the microbial community was performed using MetaPhlAn 4.

### Metabolomics analysis

For untargeted metabolomics, 50 mg of freeze-dried cecal contents were homogenized in 500 μL of 80% methanol, followed by thorough vortexing. The mixture was centrifuged, and the resulting supernatants were collected for LC-MS/MS analysis. The injection volume was 10 μL. Chromatographic separation was performed in both positive and negative electrospray ionization modes. For the positive mode, mobile phase A consisted of 0.1% formic acid in water, and mobile phase B was methanol. For the negative mode, mobile phase A was 5 mM ammonium acetate in water, and mobile phase B was methanol. The elution gradient was programmed as follows:

- 0.0–1.5 min: 2% B
- 1.5–3.0 min: linear increase from 2% to 85% B
- 3.0–10.0 min: 85% to 100% B
- 10.0–10.1 min: 100% to 2% B
- 10.1–11.0 min: 2% B
- 11.0–12.0 min: equilibration at 2% B

Quality control samples were prepared by pooling equal volumes of all individual samples to monitor analytical stability. Data processing and statistical analyses were conducted using MetaboAnalyst 6.0^64^.

### BA targeted metabolomics analysis

For metabolite extraction, 20 mg of fecal sample was mixed with 500 μL of cold methanol and 10 μL of internal standard solution. The mixture was homogenized, sonicated, and centrifuged to precipitate proteins. The resulting supernatants were collected and used for targeted metabolomics analysis by high-performance liquid chromatography-tandem mass spectrometry (HPLC-MS/MS). HPLC-MS/MS analysis was performed using a UHPLC (Waters Ltd.) coupled to a 5500 QTRAP mass spectrometer (AB SCIEX, USA). Chromatographic separation of BAs was carried out on an ACQUITY UPLC BEH C18 column (1.7 μm, 2.1 × 100 mm, Waters Ltd.). Quantitative data acquisition was conducted in MRM mode. Quality control samples, prepared by pooling aliquots of all test samples, were inserted throughout the analytical sequence to assess instrument stability and reproducibility. Data acquisition and quantification were performed using MultiQuant software.

### Flow cytometry

For analysis of macrophage populations, two cell sources were used: peritoneal lavage cells and bone marrow-derived macrophages (BMDMs). Cells were first incubated with Zombie NIR™ Fixable Viability Kit (423105, Biolegend, USA) for 20 min in the dark to exclude dead cells. After washing, Fc receptors were blocked by incubation with anti-CD16/32 antibody (E-AB-F0997A, Elabscience, China) for 15 min at room temperature. Cell staining was then performed using the following fluorescence-conjugated antibodies: FITC-conjugated anti-CD11b (101206, Biolegend, USA), PE-conjugated anti-F4/80 (111704, Biolegend, USA), APC-conjugated anti-CD86 (105012, Biolegend, USA), and BV421-conjugated anti-CD206 (141717, Biolegend, USA). Following staining, cells were resuspended in Cell Staining Buffer (420201, Biolegend, USA) and analyzed on a BD LSRFortessa™ flow cytometer (BD Biosciences, USA). Data were processed and analyzed using FlowJo software (v.10.10.0, Tree Star Inc., USA). A detailed, step-by-step visualization of the gating strategy has been shown in Supplementary Fig. 6.

### Enzyme-linked immunosorbent assays (ELISA)

Cytokine levels in cell culture supernatants and mouse serum were measured using commercial ELISA kits for TNF-α (EMC102a.96, Neobioscience Technology Co, Ltd., China), IL-1β (EMC001b.96), IL-6 (EMC004.96), IL-10 (EMC005.96), and MCP-1 (EMC113.96), following the manufacturer’s instructions. All assays were performed at room temperature using a sandwich-based ELISA format. Absorbance was measured at 450 nm using a microplate reader. Cytokine concentrations were calculated based on standard curves generated from serial dilutions of known standards.

### Hematoxylin and eosin (H&E)

Ectopic endometrial lesions and mouse colon tissues collected from mice were fixed in 4% paraformaldehyde at 4 °C for 48 h, dehydrated through a graded ethanol series, embedded in paraffin, and sectioned at a thickness of 4 μm. Tissue sections were stained with hematoxylin and eosin (H&E) following a previously described protocol^65^. The lesion area was determined as previously reported^66^. Briefly, for each endometriotic lesion, the longest axis (*X*) and the perpendicular width (*Y*) were measured using digital pathology software, and the area was calculated as *X* × *Y*. For the purpose of this study, the resulting area value was then divided by 1,000,000 to facilitate graphical presentation and recorded as the lesion score.

### Western blot analysis

Proteins were extracted from bone marrow-derived macrophages (BMDMs) using RIPA lysis buffer (FD009, Fudebio, Hangzhou, China) supplemented with a protease and phosphatase inhibitor cocktail (K1015A, APExBIO, USA). Protein concentrations were determined, and equal amounts of total protein were subjected to SDS-PAGE and transferred to PVDF membranes. Western blot was performed as previously described in ref. ^65^. The membranes were incubated with the following primary antibodies: TGR5 (1:3000, ab72608, Abcam, UK), PPARγ (1:1000, T58124S, Abmart, China), GPR132 (1:500, TP72375, Abmart, China) andβ-actin (1:5000, 66009-1-Ig, Proteintech). After incubation with appropriate HRP-conjugated secondary antibodies, bands were developed using enhanced chemiluminescence substrate for 1 min. Signals were visualized using the Alliance Q9 Advanced imaging system (UVITEC, Cambridge, UK) and quantified using ImageJ software. Protein expression levels were normalized to β-actin.

### RT-qPCR analysis

Quantitative real-time PCR (qPCR) was performed using PowerUp SYBR Green Master Mix (A25742, Thermo Fisher Scientific, USA) on a QuantStudio 6 Flex Real-Time PCR System (Thermo Fisher Scientific, USA). Full primer sequences for target genes are provided in Supplementary Table 2. Bacterial *16S* rDNA and eukaryotic *18S* rRNA were used as internal reference genes. Relative gene expression was calculated using the 2^−ΔΔCt^ method after normalization to the corresponding reference gene.

### Cell Culture and Treatment

Human immortalized endometriosis cell line (12Z) was obtained from Wuhan Pricella Biotechnology and cultured in the supplier-recommended medium. Cells were maintained at 37 °C in 5% CO₂.

BMDMs were generated from 6–8-week-old female BALB/c mice. Bone marrow was flushed from femurs and tibias using PBS, and single-cell suspensions were prepared by passing the cells through a 70 μm cell strainer. Cells were seeded into 6-well plates at a density of 1 × 10⁶ cells/mL in RPMI 1640 medium supplemented with 10% fetal bovine serum (FBS), 1% penicillin-streptomycin, and 20 ng/mL macrophage colony-stimulating factor (M-CSF; HY-P7085, MCE, USA). Medium was replaced every 48 hours, and cells were maintained at 37 °C in 5% CO₂.

On day 6, cells were polarized under the following conditions for 24 hours:

- 20 ng/mL M-CSF (control),
- 20 ng/mL IFN-γ (315-05, Peprotech, USA) plus 100 ng/mL lipopolysaccharide (LPS; 297–473–0, SIGMA, USA) for M1 polarization,
- 20 ng/mL IL-4 (214–14, Peprotech, USA) for M2 polarization.

On day 7, BMDMs were treated with 7-KLCA at concentrations of 0 μM, 5 μM, 10 μM, or 20 μM for 24 h. After treatment, culture supernatants were collected and stored at −80 °C for subsequent assays. Cells were washed with sterile PBS and processed for downstream experiments.

Based on our findings, 10 μM was identified as the most effective concentration and was therefore selected for all further investigations. To evaluate the interaction between 7-KLCA and TGR5 signaling, a separate experiment was performed. M2 macrophages were pretreated with 5 μM of the TGR5 antagonist SBI-115 (HY-111534, MCE, USA) for 2 h^67^. The medium was then replaced, and cells were treated with 10 μM 7-KLCA for an additional 22 h. Cells were collected for downstream analysis.

### RNA extraction and transcriptome analysis

Total RNA was extracted from cultured cells using TRIzol reagent according to the manufacturer’s instructions. Following phase separation with chloroform, the aqueous phase containing RNA was collected after centrifugation (12,000 rpm, 15 min, 4 °C). RNA was then precipitated with isopropanol, washed with 75% ethanol, and resuspended in RNase-free water. Complementary DNA (cDNA) was synthesized using a commercial reverse transcription kit (CW2020M, CWBIO, China). PCR amplification products were purified, and library quality was assessed using an Agilent 2100 Bioanalyzer. Libraries were sequenced on the Illumina NovaSeq platform. Gene expression levels were quantified as fragments per kilobase of transcript per million mapped reads (FPKM). Sequence alignment was performed using Hisat2 (v2.0.5), and read counts were obtained with featureCounts (v1.5.0-p3). Downstream analyses were conducted in R (v4.4.3). Differentially expressed genes (DEGs) were identified using the DESeq2 package (v1.46.0)^68^, with significance thresholds set at an adjusted *p*-value < 0.05 and absolute log2 fold change ≥ 1. Kyoto Encyclopedia of Genes and Genomes (KEGG) pathway enrichment analysis was performed in R, and visualization of enriched pathways was generated using ggplot2 (v3.3.3) and OmicStudioKits (v3.49.0).

### Analysis of publicly available single-cell RNA-seq data (GSE213216)

Publicly available single-cell RNA-seq data from endometrial samples (accession GSE213216) were downloaded from the Gene Expression Omnibus (GEO). The dataset includes samples from endometrioma, eutopic endometrium, endometriosis, unaffected ovary, and non-endometriosis control tissues.

Raw count matrices were processed using the Seurat package (v4.3.0) in R (v4.2.0). Cells were filtered based on the number of detected genes, total counts, and mitochondrial gene percentage. Specifically, cells with fewer than 200 detected genes or with mitochondrial content exceeding 20% were excluded.

Data were normalized using the LogNormalize method with a scale factor of 10,000. Highly variable features were identified using the FindVariableFeatures function with the “vst” selection method. Principal component analysis (PCA) was performed, and the first 30 principal components were used for downstream analysis. UMAP (Uniform Manifold Approximation and Projection) dimensionality reduction was performed using the RunUMAP function with default parameters (n.neighbors = 30, min.dist = 0.3) to visualize cell populations across different tissue types.

Immune cell subsets were annotated based on the expression of canonical marker genes: cDC1 (CLEC9A, CADM1), cDC2 (CD1C, CLEC10A), DC3 (LAMP3, CCR7), monocytes (CD14, FCGR3A), macrophages (CD68, CD163), M1 macrophages (IL1B, CCL3), M2 macrophages (CD163, MRC1), and neutrophils (CSF3R, S100A8). Normalized marker gene expression was visualized using heatmaps with Z-score transformation, where red indicates high expression and blue indicates low expression.

The expression of bile acid-related genes, including GPBAR1 (TGR5), was examined across different histopathological tissue types and immune cell subsets. Average gene expression levels were calculated for each cell type and tissue condition, and visualized as heatmaps. GPBAR1 expression was specifically examined across all tissue types (endometrioma, endometrium, endometriosis, unaffected ovary, and non-endometriosis controls) using feature plots overlaid on UMAP projections.

### Molecular docking analysis

The binding conformations of 7-KLCA with the TGR5 receptor were analyzed using AutoDock4. The crystal structure of TGR5 (PDB ID 7BW0) was prepared by removing water molecules and non-essential ligands, and adding counter-ions as needed. A grid box was defined and adjusted to fully encompass the ligand-binding pocket. The structure of 7-KLCA (CID 444262) was retrieved from the PubChem database and prepared for docking using AutoDock4 tools. Molecular docking was then performed, and docking scores were used to evaluate the binding affinity between 7-KLCA and TGR5.

### Cellular thermal shift assay (CETSA)

HEK 293 T cells were transfected with the TGR5-Flag plasmids using Lipofectamine 3000 (L3000001, Thermo Fisher, USA). Following transfection, the cells were lysed with NP-40 buffer (HY-Y1884, MCE, USA) and subjected to three freeze-thaw cycles in liquid nitrogen. The lysates were then centrifuged at 15,000 × *g* for 15 min at 4 °C. The resulting supernatants were aliquoted into eight PCR tubes and incubated with 10 μM 7-KLCA or vehicle (DMSO) for 2 hours at room temperature. Each aliquot was subsequently heated for 3 min at a specific temperature in a gradient (35 °C, 40 °C, 45 °C, 50 °C, 55 °C, 60 °C, 70 °C, and 80 °C), followed by centrifugation to collect the supernatant. Protein stability was assessed by Western blot according to standard procedures.

### Surface plasmon resonance (SPR) analysis

Binding kinetics of 7-KLCA to immobilized TGR5 protein were measured at 25 °C on a BIAcore 1 K (Cytiva) using CM5 chips. TGR5 in 10 mM sodium acetate (pH 5.0) was covalently immobilized on EDC/NHS-activated surfaces (200 mM EDC/50 mM NHS; 10 μL/min, 7 min). Reference flow cells were identically prepared but immobilized with PBS (pH 5.0). All surfaces were blocked with 1 M ethanolamine (10 μL/min, 7 min).

Serially diluted 7-KLCA in PBS was injected (10 μL/min, 150 s association) followed by regeneration with 10 mM glycine-HCl (pH 2.0; 10 μL/min, 5 min). Data collected via Biacore Insight (v2.0) were reference-subtracted and globally fitted to a 1:1 Langmuir model using BIAcore 1 K Evaluation Software to determine *K*_D_, *K*_a_, and *K*_d_. Figures were prepared in Origin 7 (v7.0552).

### Efferocytosis assay

Adherent macrophages were labeled with DiI cell-labeling solution (C1991S, Beyotime, China) for 15 min at 37 °C, followed by nuclear counterstaining with Hoechst 33342 (C1028, Beyotime, China). In parallel, 12Z cells were induced to undergo apoptosis by ultraviolet irradiation for 15 min, followed by a 6-h incubation at 37 °C in a humidified atmosphere containing 5% CO₂ to allow apoptotic progression. Early apoptotic cells (ACs) were identified as annexin V-positive populations by flow cytometry. Prior to co-culture, ACs were resuspended in complete culture medium and added to macrophages at a 5:1 AC-to-macrophage ratio. After 3 h of co-culture, non-engulfed ACs were removed by two gentle washes with PBS. Internalized apoptotic cells were then detected by TUNEL staining (C1086, Beyotime, China) for 1 h at 37 °C. The experiment was independently repeated three times. For each replicate, three random microscopic fields per condition were captured. Fluorescence images were acquired using a Leica TCS SP8 fluorescence microscope. Efferocytic efficiency was calculated as the fraction of macrophages with at least one efferosome.

### Statistical analysis

Statistical analyses were performed using GraphPad Prism software (v10.3.1). Data normality was assessed using the Shapiro–Wilk test. For normally distributed data, two-group comparisons were performed using unpaired Student’s *t*-test. One-way and two-way analyses of variance (ANOVA) followed by Bonferroni’s post hoc test were used for comparisons among multiple groups. For non-normally distributed data, the Mann–Whitney *U* test was used for two-group comparisons, while the Kruskal-Wallis test followed by Dunn’s post hoc test was used for multiple group comparisons. A *p*-value of < 0.05 was considered statistically significant.

## Supplementary information

Supplementary_Information

## Data Availability

The data that support the findings of this study are openly available in National Microbiology Data Center (NMDC) at https://nmdc.cn/resource/genomics/project/detail/NMDC10019913, reference number NMDC20394524.
