# Supplementary material for: Parabacteroides goldsteinii and its metabolite 7-KLCA attenuate endometriosis via TGR5 to reprogram macrophages by modulating the PPARγ/GPR132 axis
Source: NPJ Biofilms Microbiomes. 2026 May 22;12:153. doi: 10.1038/s41522-026-01017-4 (PMC13473097; doi:10.1038/s41522-026-01017-4)

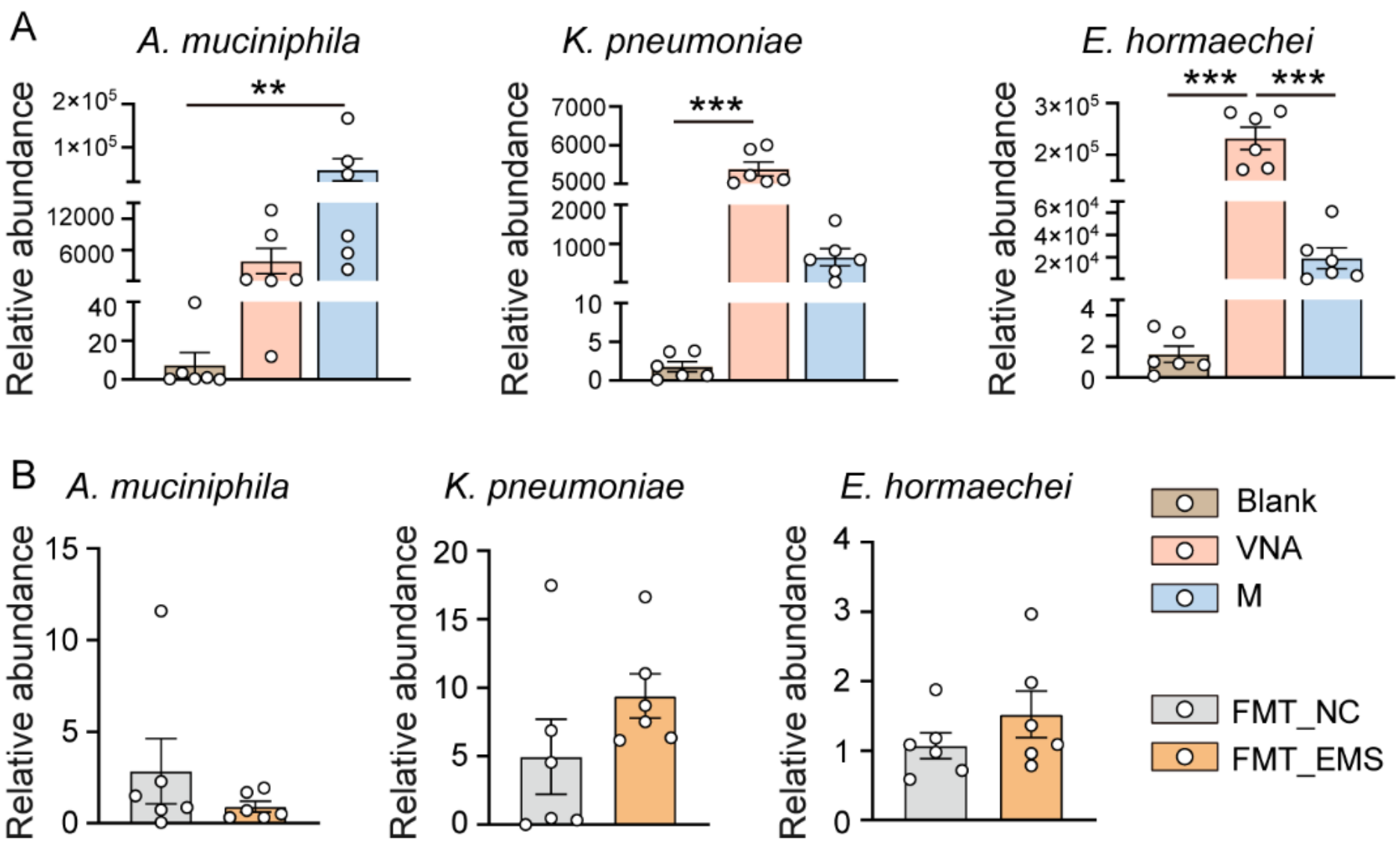

Supplementary fig 1. Effects of antibiotic treatment and fecal microbiota transplantation on the relative abundance of *A. muciniphila*, *K. pneumoniae*, and *E. hormaechei*. (A) Comparative analysis of the relative abundance of *A. muciniphila, K. pneumoniae,* and *E. hormaechei* across Blank, VNA, and M groups (n = 6/group). Statistical differences were analyzed by Kruskal-Wallis test followed by Dunn's test (*A. muciniphila* and *K. pneumoniae*) or one-way ANOVA followed by Bonferroni's post-hoc test (*E. hormaechei*), respectively. (B) The relative abundance of *A. muciniphila*, *K. pneumoniae,* and *E. hormaechei* between the FMT_NC and FMT_EMS groups (n = 6/group). Statistical differences were analyzed by the two-sided Mann-Whitney U test (*A. muciniphila*) or a two-tailed *t*-test (*K. pneumoniae* and *E. hormaechei*), respectively. Data are presented as mean ± SEM. *$p < 0.05$, **$p < 0.01$, ***$p < 0.001$.

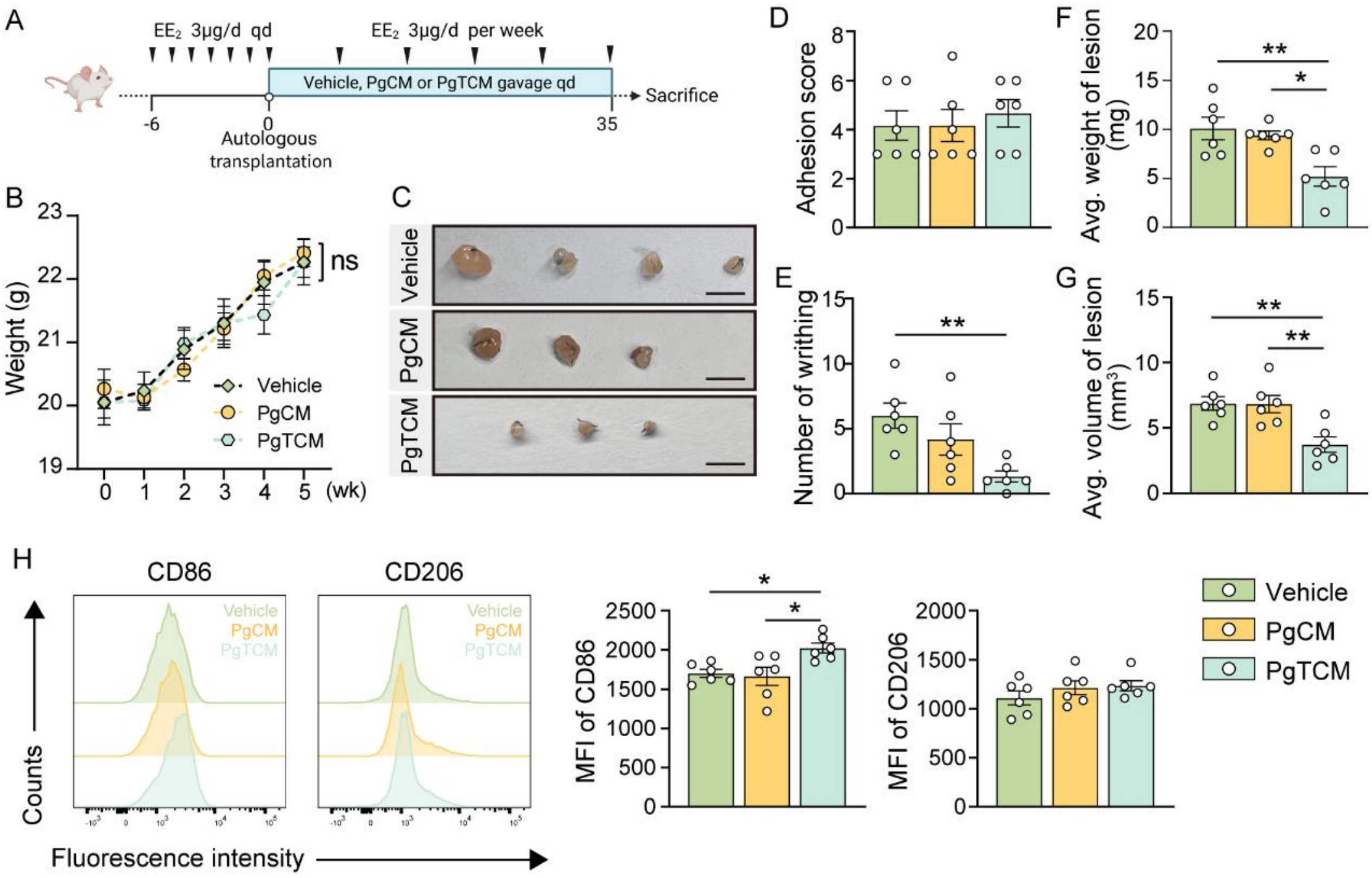

Supplementary fig 2. TCDCA supplementation in Pg medium mitigate EMS phenotypes. (A) The mice were administered EE_2 via daily injections for 7 days, followed by weekly injections thereafter. They

were then allocated to three oral gavage regimens: Vehicle, PgCM, and PgTCM. (B) Body weight changes (n = 6/group), as determined using a two-way ANOVA followed by Bonferroni's post-hoc test. (C) Ectopic endometriotic lesion representative images (scale bar = 5 mm). (D) Adhesion scores (n = 6/group). (E) The number of writhing (n = 6/group). (F) The average weight of lesions (n = 6/group). (G) The average volume of lesions (n = 6/group). (H) The MFI of CD86 and CD206 was measured by flow cytometry in macrophages derived from peritoneal lavage (n = 6/group). Data was shown in mean ± SEM. The differences between groups were compared using one-way ANOVA followed by Bonferroni's post-hoc test for multiple comparisons in panels D, F, G, and H. For panel E, data was analyzed by Kruskal-Wallis test followed by Dunn's test. *$p < 0.05$, **$p < 0.01$, ***$p < 0.001$. TCDCA, taurochenodeoxycholic acid; Pg, *Parabacteroides goldsteinii*; EE_2, estradiol benzoate; PgCM, Pg culture medium; PgTCM, Pg culture medium supplemented with TCDCA; ns: not significant; MFI, mean fluorescence intensity.

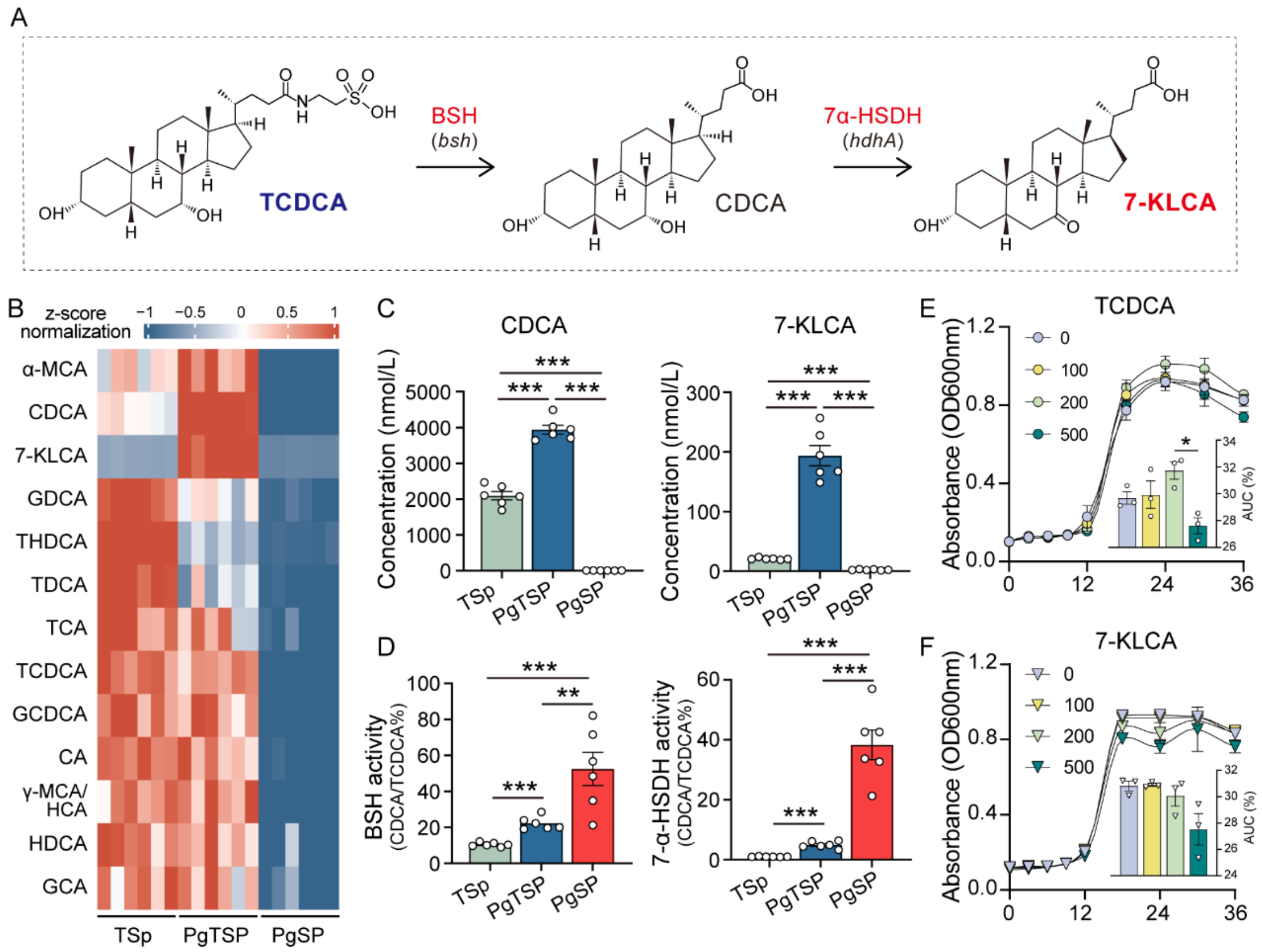

Supplementary fig 3. *Pg* converts TCDCA into 7-KLCA. (A) The conversion of TCDCA to 7-KLCA involves taurine de-conjugation, and 7α-dehydroxylation, catalyzed by BSH and 7α-HSDH enzymes. (B) Heat maps of bile acid profiles of TSp, PgTSP and PgSP groups. (C) The concentration of CDCA and 7-KLCA (n = 6/group). (D) The activity of BSH and 7α-HSDH enzymes (n = 6/group). (E) The absorbance of Pg (OD600 nm) under TCDCA treatment at various concentrations in vitro (0 μM, 100 μM, 200 μM and 500 μM). The bottom right panel displays the AUC for each TCDCA dosage (n = 3/group). (F) The absorbance of Pg (OD600 nm) under 7-KLCA treatment at various concentrations in vitro (0 μM, 100 μM, 200 μM and 500 μM). The bottom right panel displays the AUC for each 7-KLCA dosage (n = 3/group). Data was presented as mean ± SEM. Spearman's correlation test was used for statistical analysis in panel B. The differences between groups were compared using one-way ANOVA followed by Bonferroni's post-

hoc test for multiple comparisons in panel C, D, E, and F. *p < 0.05, **p < 0.01, ***p < 0.001. TCDCA, taurochenodeoxycholic acid; CDCA, chenodeoxycholic acid; 7-KLCA, 7-ketolithocholic acid; BSH, bile salt hydrolase; 7α-HSDH, 7α-hydroxysteroid dehydrogenase; AUC, area under the curve; Pg, *Parabacteroides goldsteinii*; TSp, medium supplemented with TCDCA; PgSP, Pg medium supernatant; PgTSP, Pg medium supernatant supplemented with TCDCA.

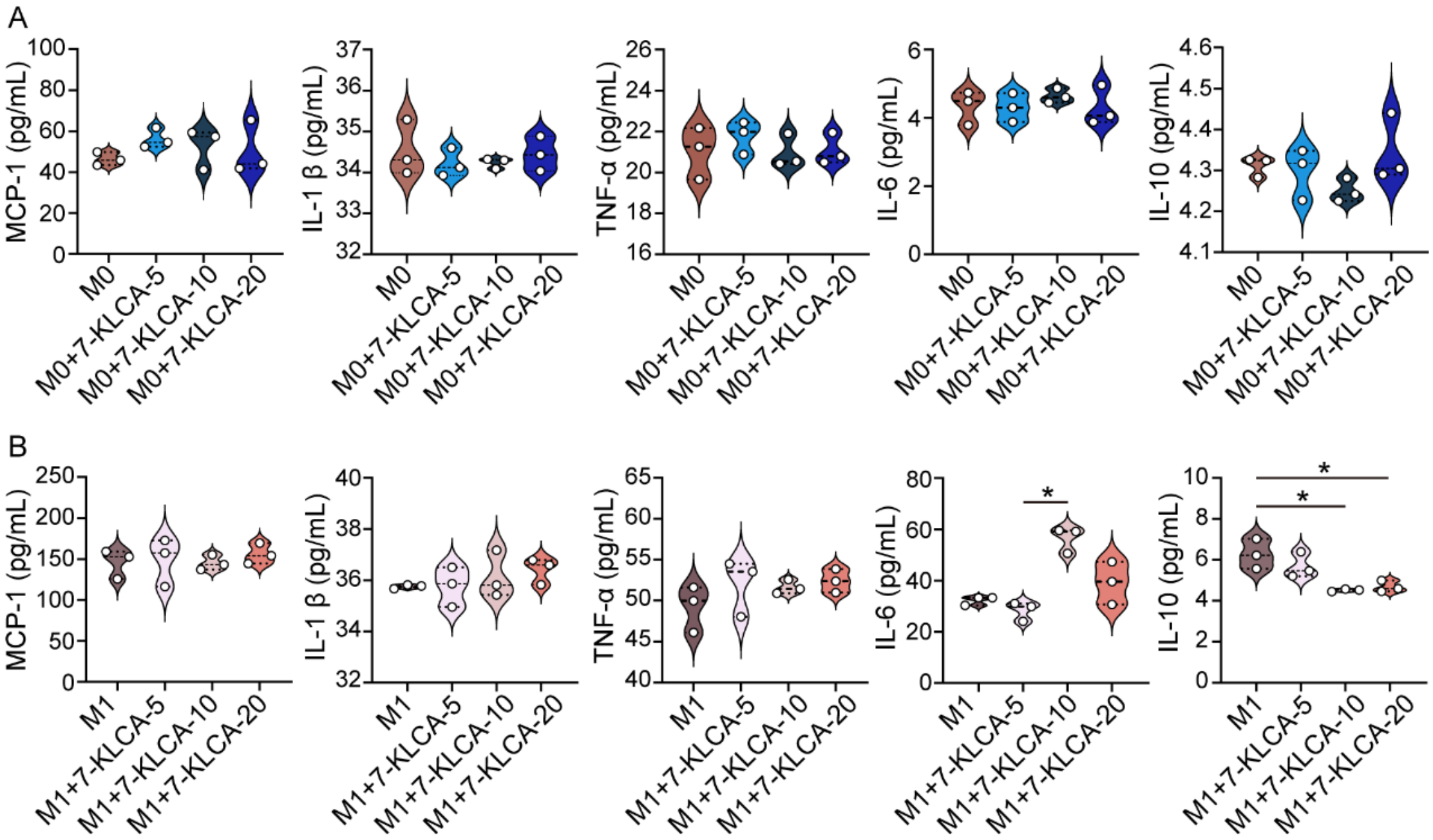

Supplementary fig 4. No response of M0 and M1 macrophages to 7-KLCA stimulation. (A-B) ELISA quantification of inflammatory cytokines (MCP-1, IL-1β, TNF-α, IL-6, IL-10) in culture medium supernatants from M0 and M1 macrophages treated with 7-KLCA at concentrations of 0 μM, 5 μM, 10 μM, and 20 μM (n= 3/group). Data was shown in median and IQR. The differences between groups were compared using one-way ANOVA followed by Bonferroni's post-hoc test for multiple comparisons. *p < 0.05, **p < 0.01, ***p < 0.001. 7-KLCA, 7-ketolithocholic acid.

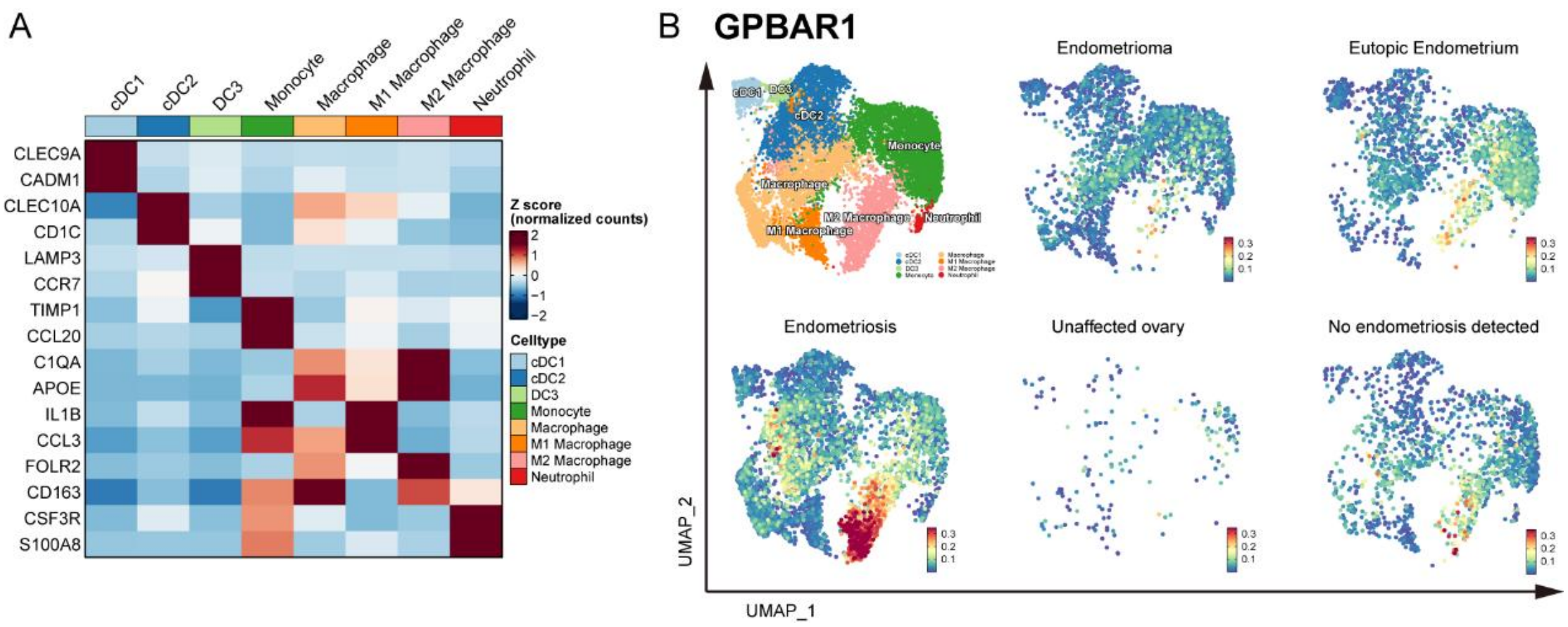

Supplementary fig 5. Transcriptional profiling of immune cells and GPBAR1 expression in endometriosis-associated tissues. (A) Normalized expression of marker genes for immune cell subsets

identified by single-cell RNA sequencing. Color gradient indicates Z scores (-2 to 2), with red (high) and blue (low) expression. (B) UMAP shows GPBAR1 expression across different tissue types: endometrioma, endometrium, endometriosis, unaffected ovary and non-endometriosis controls.

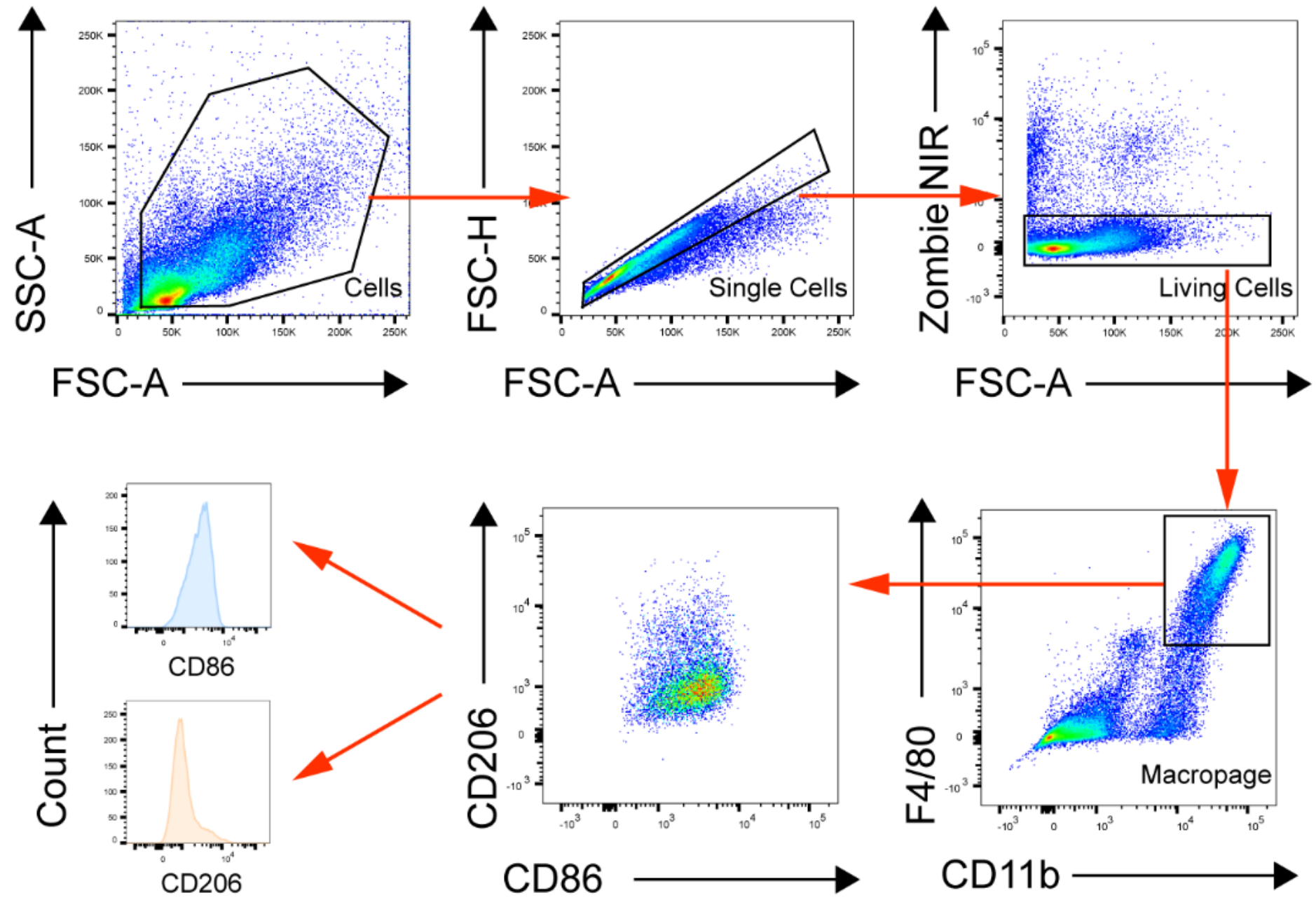

Supplementary fig 6. Gating strategy for identifying macrophages and assessing CD206 and CD86 MFI by flow cytometry. Representative plots depict the sequential gating procedure: cells were gated from debris, single cells were selected by excluding doublets, viable cells were identified, and macrophages were isolated as the $F4/80^{+}CD11b^{+}$ population. The Median Fluorescence Intensity (MFI) of CD206 and CD86 was subsequently analyzed within this defined macrophage population.

Supplementary Table 1. Clinical characteristics of the FMT donors

		NC Group (n=5)	EMS Group (n=5)	*P*
Basic information	Age (y)	31.4±6.54	33.6±5.41	0.578
	Weight (kg)	56.0±13.5	54.4±2.79	0.802
	Height (cm)	158.0±5.94	158.0±6.73	>0.999
	BMI (kg/m^2)	22.1±3.52	21.7±1.67	0.843
	Gravidity	0 (0,2.5)	2 (0,2.5)	0.921
	Parity	0 (0-1)	0 (0-2)	0.683
	Menstrual cycle (d)	30.2±2.77	28±2.12	0.197
	Menstrual period (d)	7 (4,7)	7 (5,7)	0.722
Routine blood test	WBC (×10^9)	6.61±2.02	8.84±2.97	0.201
	Hb (g/L)	108±9.02	115±16.7	0.420
	PLT (×10^9)	267±42.1	261±33.1	0.808
	PT (s)	12.9±0.921	13.6±0.680	0.261
Liver and renal function	AST (U/mL)	18.6±4.45	17.2±2.28	0.549
	ALT (U/mL)	14.4±6.841	15.2±6.686	0.856
	ALB (g/L)	42.6±3.61	42.2±3.81	0.849
	Cr (mmol/L)	51 (51, 64)	58 (49, 63.5)	0.921
Disease severity	Stage	-	Stage Ⅱ (n = 1)<br>Stage III (n = 4)	-
	ASRM score	-	21 (20, 23)	-
	CA-125 (U/L)	13.6 (10.1, 14.8)	89.8 (35.1, 506)	*0.008
	HE4（pmol/L）	47.3±14.2	60.8±17.5	0.217

Data was presented as median and IQR or mean ± SD based on normality tests. BMI, body mass index; WBC, white blood cell; Hb, hemoglobin, PLT, platelet; AST, aspartate transaminase; ALT, alanine aminotransferase; ALB, albumin; Cr, creatinine; Stage, disease stage identified by American Society for Reproductive Medicine; ASRM score, scoring system of American Society for Reproductive Medicine; CA-125, carbohydrate antigen 125; HE4, human epididymis protein 4.

Supplementary Table 2. Sequences of the real time PCR primers used in this study

PCR primers		
18s	Forward Primer:	5′-AGTCCCTGCCCTTTGTACACA-3′
	Reverse Primer:	5′-CGATCCGAGGGCCTCACTA-3′
Fxr	Forward Primer:	5′-CTGGCATCTATGAACTCAGGC-3′
	Reverse Primer:	5′-CCATTCGCGGCTTCTTTGT-3′
Gpbar1	Forward Primer:	5′-CCTGGAACTCTGTTATCGCTCA-3′
	Reverse Primer:	5′-GCACTCGTAGACACCTTTGGG-3′
Car	Forward Primer:	5′-GGGCCACAGGCTATCATTTCC-3′
	Reverse Primer:	5′-CCTCATGCCAACATTTAGACACT-3′
Vdr	Forward Primer:	5′-GAATGTGCCTCGGATCTGTGG-3′
	Reverse Primer:	5′- ATGCGGCAATCTCCATTGAAG-3′
Smpd3	Forward Primer:	5′-CCTGACCAGTGCCATTCTTT-3′
	Reverse Primer:	5′-AGAAACCCGGTCCTCGTACT-3′
Pparα	Forward Primer:	5′-ACCACTACGGAGTTCACGCATG-3′
	Reverse Primer:	5′-GAATCTTGCAGCTCCGATCACAC-3′
Mdr1	Forward Primer:	5′-GGTGGTGTCATTGTGGAGCAAG-3′
	Reverse Primer:	5′-GCATCAGTGTCACTCTGGGATC-3′
Mrp4	Forward Primer:	5′-CACTCAGGAAACGAACCTTCTCC-3′
	Reverse Primer:	5′-TTGCACTGCCTGCGTGTTCTCT-3′
Stab1	Forward Primer:	5′- GGACTGCCGCTATGAGTTG-3′
	Reverse Primer:	5′- GCAGACTGAACCGCTGAAGTT-3′
Gpr132	Forward Primer:	5′-CCTGCAAGGTGACTGCTTACA-3′
	Reverse Primer:	5′-GAAGAATCACACACGCAGAAATG-3′
C1qa	Forward Primer:	5′-TTCGGCAGAACCCAATGACG-3′
	Reverse Primer:	5′-TGGTATGGACTCTCCTGGTTG-3′
Dusp4	Forward Primer:	5′-TCCCCGTCGAAGACAACCA-3′
	Reverse Primer:	5′-CTTTACTGCGTCGATGTACTCG-3′
Sphk1	Forward Primer:	5′-ACTGATACTCACCGAACGGAA-3′
	Reverse Primer:	5′-CCATCACCGGACATGACTGC-3′
Pecam1	Forward Primer:	5′-ACGCTGGTGCTCTATGCAAG-3′
	Reverse Primer:	5′-TCAGTTGCTGCCCATTCATCA-3′
Pparg	Forward Primer:	5′-GCAGCTACTGCATGTGATCAAGA-3′
	Reverse Primer:	5′-GTCAGCGGGTGGGACTTTC-3′
CD36	Forward Primer:	5′-AGATGACGTGGCAAAGAACAG-3′
	Reverse Primer:	5′-CCTTGGCTAGATAACGAACTCTG-3′
Mfge8	Forward Primer:	5′-AGATGCGGGTATCAGGTGTGA-3′
	Reverse Primer:	5′-GGGGCTCAGAACATCCGTG-3′
Dnmt3a	Forward Primer:	5′-GATGAGCCTGAGTATGAGGATGG-3′
	Reverse Primer:	5′-CAAGACACAATTCGGCCTGG-3

Supplementary Table 2 (continued)

16s	Forward Primer:	5′-GTGSTGCAYGGYTGTCGTCA-3′
	Reverse Primer:	5′-ACGTCRTCCMCACCTTCCTC-3′
Pg	Forward Primer:	5′-GAATAAAGTGAGGAACGTGTT-3′
	Reverse Primer:	5′-AACTTTCACCGCTGACTTAATTA-3′
Am	Forward Primer:	5′-AGCCTGAACGCATTATCCGC-3′
	Reverse Primer:	5′-AGGGCAGTTCTTCCAGCGTA-3′
Kp	Forward Primer:	5′-GCGGACGGGTGAGTAATGTC-3′
	Reverse Primer:	5′-AGCCGTTACCCCACTACTA-3′
Eh	Forward Primer:	5′-AACTGTCAGGGTTTGCGC-3′
	Reverse Primer:	5′-CAAACAGCGCCACGTTAT-3′

Supplementary Table 3. Clinical characteristics of healthy controls and patients with endometriosis

		Healthy Controls (n=12)	Endometriosis (n=16)	*P*
Basic information	Age (y)	36.9±7.6	34.9±6.3	0.457
	Weight (kg)	54.4±8.6	56.7±7.2	0.453
	Height (cm)	160.1±3.7	161.0±5.7	0.614
	BMI (kg/m2)	21.1 ± 2.9	21.9±3.1	0.492
Serum markers	CA-125 (U/L)	15.1 (10.3, 18.6)	16.7 (10.0,28.4)	0.534
	HE4（pmol/L）	30.5 (23.0, 32.8)	57.2 (22.0, 82.1)	**0.006**

Data are presented as mean±SD or median (25th-75th percentile) depending on data distribution. Age, Weight, Height, BMI: Normally distributed, analyzed by Student's t-test. CA-125, HE4: Non-normally distributed, analyzed by Mann-Whitney U test. BMI, body mass index; CA-125, carbohydrate antigen 125; HE4, human epididymis protein 4.

Supplementary fig 7. Uncropped scans of CETSA presented in Figure 7E.

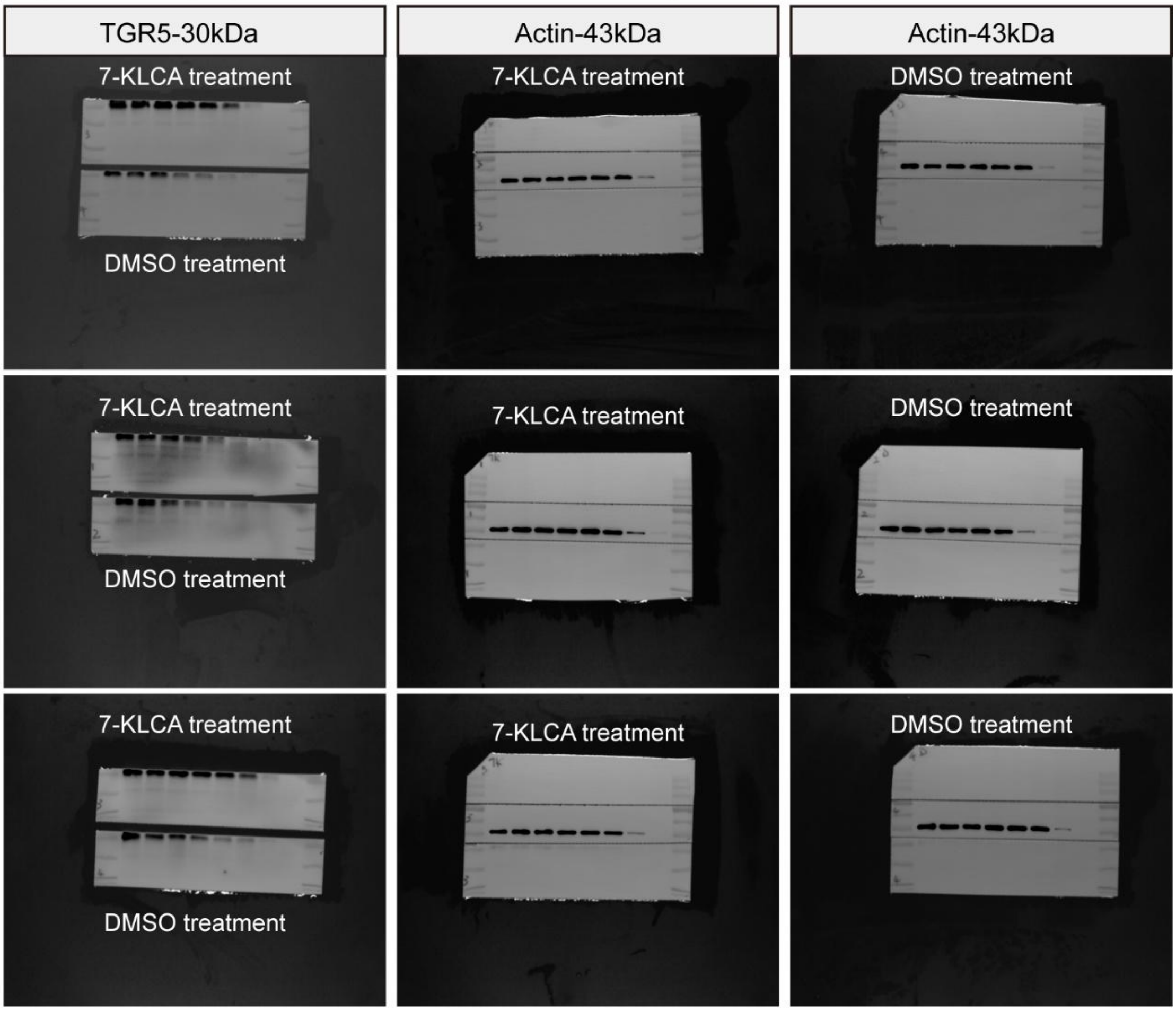

Supplementary fig 8. Uncropped scans of Western blots presented in Figure 8E.

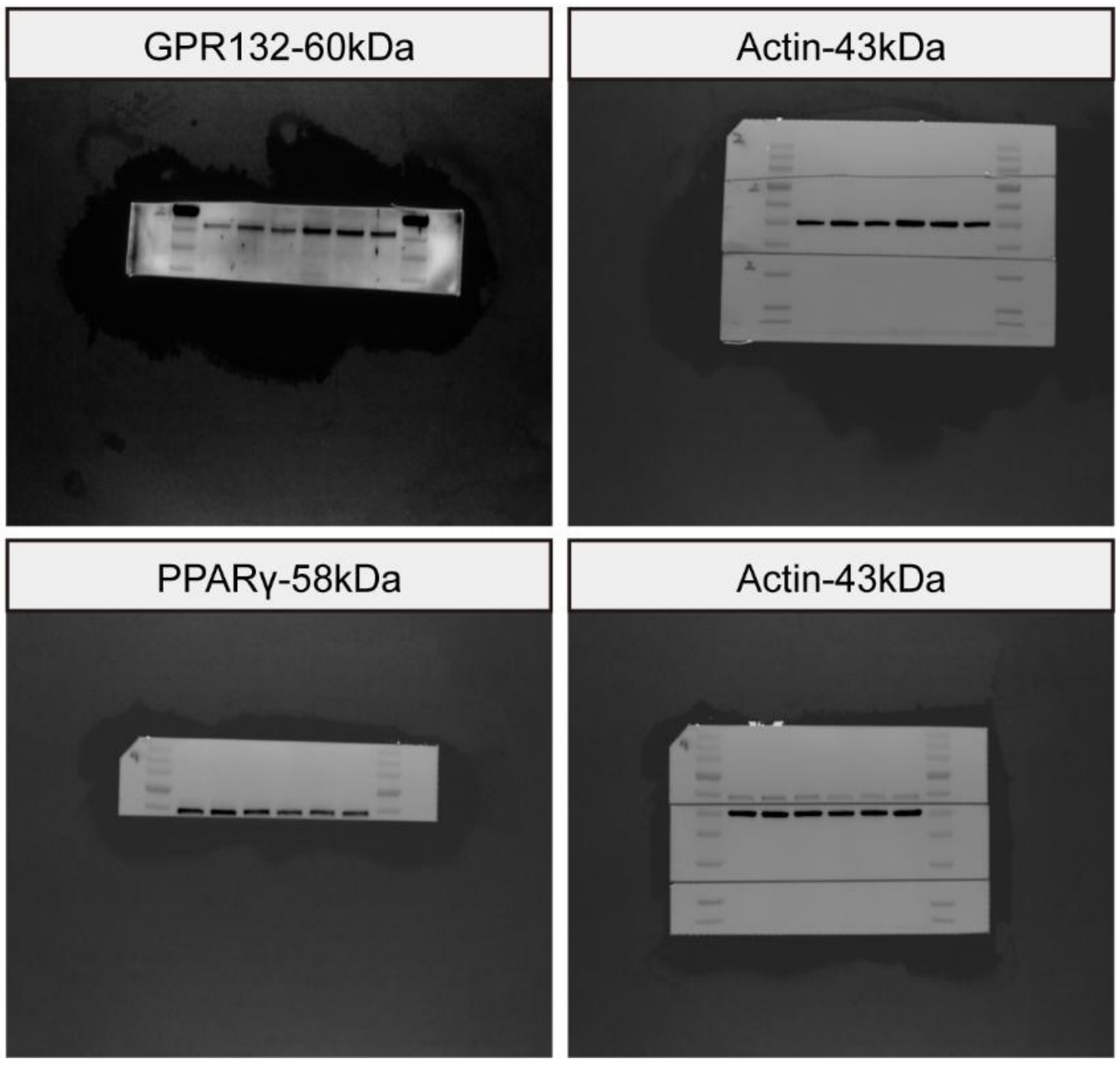

Supplement: Supplementary file 1 [file 41522_2026_1017_MOESM1_ESM.pdf]
